# High *IL1R1* expression predicts poor survival and benefit from stem cell transplant in intermediate-risk acute myeloid leukemia from the Leucegene cohort

**DOI:** 10.1186/s40364-025-00827-6

**Published:** 2025-10-23

**Authors:** Guillaume Richard-Carpentier, François Béliveau, Sandrine Lacoste, Banafsheh Khakipoor, Véronique Lisi, Michael Vladovsky, Miriam Marquis, Jean-François Spinella, Patrick Gendron, Sébastien Lemieux, Vincent-Philippe Lavallée, Guy Sauvageau, Josée Hébert

**Affiliations:** 1 Department of Medicine, Division of Medical Oncology and Hematology, Princess Margaret Cancer Centre, University Health Network, University of Toronto, 610 University Avenue, OPG Building, 700 U, Toronto, ON M5G 2M9 Canada; 2 Department of Medicine, Division of Medical Oncology and Hematology, Temerty Faculty of Medicine, University of Toronto, Toronto, ON Canada; 3 Quebec Leukemia Cell Bank, Centre de Recherche de L’Hôpital Maisonneuve-Rosemont, Montréal, QC Canada; 4 Centre Hospitalier Universitaire Sainte-Justine, Montréal, QC Canada; 5 Institute for Research in Immunology and Cancer, Université de Montréal, Montréal, QC Canada; 6 Department of Computer Science and Operations Research, Université de Montréal, Montréal, QC Canada; 7 Department of Pediatrics, Faculty of Medicine, Université de Montréal, Montréal, QC Canada; 8 Department of Medicine, Division of Hematology-Oncology and Cell Therapy, Hôpital Maisonneuve-Rosemont Université de Montréal, 5415 Boulevard L’Assomption, Montréal, QC H1T 2M4 Canada; 9 Department of Medicine, Faculty of Medicine, Université de Montréal, Montréal, QC Canada

**Keywords:** Acute myeloid leukemia, Transcriptomics, Gene expression, Predictive biomarker, Prognosis, Allogeneic stem cell transplantation

## Abstract

**Background:**

There is an unmet clinical need to identify patients with acute myeloid leukemia and intermediate-risk cytogenetics who benefit from allogeneic hematopoietic stem cell transplantation in first remission, especially among those without *FLT3*-ITD mutation.

**Methods:**

We analyzed transcriptomic data from the Leucegene cohort composed of 316 patients with acute myeloid leukemia and intermediate-risk cytogenetics who have been treated with intensive chemotherapy. We evaluated associations between gene expression and overall survival or relapse-free survival and we analyzed the interaction between gene expression and allogeneic hematopoietic stem cell transplantation to identify biomarkers that predict the benefit of stem cell transplantation in this subgroup of patients.

**Results:**

We identified high *IL1R1* expression (*IL1R1*^high^) as a prognostic and predictive marker in the Leucegene cohort. *IL1R1*^high^ (≥ 2.0 transcripts per million) was associated with older age, monocytic differentiation, higher frequency of *FLT3*-ITD and *RUNX1* mutations and lower frequency of *IDH1*/*2* and bZIP *CEBPA* mutations. Patients with *IL1R1*^high^ had lower 5-year overall survival (10% vs 38%, *p* < 0.01), and higher 5-year cumulative incidence of relapse (76% vs 59%, *p* < 0.01) than those with low *IL1R1* expression. *IL1R1*^high^ was independently associated with overall survival in multivariable analyses including age, white blood cell count at diagnosis and *NPM1*, *FLT3*-ITD, bZIP *CEBPA*, *RUNX1*, *ASXL1* and *DNMT3A* mutations (HR 1.78, *p* < 0.01). Importantly, in landmark analysis, hematopoietic stem cell transplantation in first remission significantly improved 5-year overall survival in patients with *IL1R1*^high^ (67% vs 27%, HR 0.33, *p* < 0.01), but not in patients with *IL1R1*^low^ (62% vs 54%, HR 0.72, *p* = 0.31), especially among those without *FLT3*-ITD mutation (48% vs 50%, HR 0.93, *p* = 0.85). In patients who proceeded to allogeneic hematopoietic stem cell transplantation, the 5-year overall survival was 60% in patients with *IL1R1*^high^ compared to 56% in patients with *IL1R1*^low^ confirming that the worse prognosis associated with high expression of *IL1R1* was abrogated by stem cell transplantation.

**Conclusion:**

*IL1R1* expression is a candidate marker to identify patients with intermediate-risk cytogenetics acute myeloid leukemia at high risk of relapse who benefit from allogeneic hematopoietic stem cell transplantation in first remission.

**Supplementary Information:**

The online version contains supplementary material available at 10.1186/s40364-025-00827-6.

## Background

Acute myeloid leukemia (AML) is a heterogeneous hematological malignancy of hematopoietic progenitor and stem cells with a globally poor prognosis [1–3]. The standard intensive treatment for eligible patients consists of induction chemotherapy followed by consolidation chemotherapy or allogeneic hematopoietic stem cell transplantation (HSCT) [4]. Clinical characteristics and genetic features of leukemia cells, including cytogenetic abnormalities and gene mutations, are used to select the appropriate treatment including HSCT in first remission (CR1) [4–6]. Based on the European LeukemiaNet (ELN) risk classification, eligible patients with adverse-risk AML should undergo HSCT in CR1 whereas most patients with favorable-risk AML should be treated with consolidation chemotherapy without HSCT [4, 7]. In patients with intermediate-risk cytogenetics (IRC), HSCT in CR1 is generally recommended based on non-randomized studies showing improvement in overall survival (OS) [8–11]. However, in this subgroup of patients, the benefit from HSCT is of lesser magnitude compared to patients with adverse-risk AML and accordingly, lower thresholds for estimated risks of transplant-related morbidity and mortality are deemed acceptable [7, 12–14]. The role of HSCT in CR1 for patients with IRC AML has recently been further questioned with the results of a prospective randomized trial showing no benefit in OS with HSCT compared to consolidation chemotherapy [15].

The mutational profile of IRC AML helps to identify patients who benefit from HSCT, notably those with *FLT3*-ITD mutations (*FLT3*-ITD +) [10, 16–18]. In contrast, HSCT is generally not recommended in patients with bZIP *CEPBA* or with *NPM1* mutation without *FLT3*-ITD mutation (*NPM1* +/*FLT3*-ITD–) [4, 19–21]. However, despite being classified as favorable-risk AML, about half of patients with *NPM1* +/*FLT3*-ITD– relapse and would likely benefit from HSCT in CR1. Furthermore, a substantial proportion of patients are negative for *FLT3*-ITD, *NPM1* and bZIP *CEBPA* mutations and the benefit of HSCT in CR1 in patients with myelodysplasia-related gene mutations (MRGM) remains uncertain. Therefore, additional biomarkers to identify at diagnosis the patients with IRC AML who benefit from HSCT in CR1 would be extremely valuable.

With the aim of discovering biomarkers to predict the benefit of HSCT, we analyzed whole transcriptome sequencing data in the Leucegene *de novo* IRC AML cohort composed of 316 patients treated with intensive chemotherapy. We report herein the identification of *IL1R1* as a gene expression biomarker that is independently associated with adverse clinical outcomes and identifies patients with IRC AML who benefit from HSCT in CR1, especially among those without *FLT3*-ITD mutation.

## Methods

### Patient cohort

The Leucegene *de novo* IRC AML cohort includes 316 newly diagnosed patients treated with intensive chemotherapy with bone marrow (BM) or peripheral blood (PB) cells collected at diagnosis by the Quebec Leukemia Cell Bank between 2001 and 2018 (Table 1 and Additional file 1: Table S1). Cytogenetics data was obtained from standard karyotype and fluorescent in situ hybridization and intermediate-risk was defined following the Medical Research Council and ELN 2022 classifications [4, 5].

**Table 1:** Characteristics of patients according to *IL1R1* expression

Characteristic	Total cohort (n = 316)	*IL1R1*^low^ (n = 193)	*IL1R1*^high^ (n = 123)	*p* value
Age at Dx — median [range]	56 [20–78]	54 [20–78]	59 [21–76]	< 0.01
Age ≥ 60 years old	122 (39)	63 (33)	59 (48)	< 0.01
Sex (male) — n (%)	167 (53)	103 (53)	64 (52)	0.91
WBC at Dx (× 10^9^/L)	37.0 [0.7–375.6]	31.4 [0.7–361.2]	43.7 [1.3–375.6]	0.06
WBC ≥ 50 × 10^9^/L*	130/314 (41)	75/191 (39)	55/123 (45)	0.40
PB blasts at Dx (%)	70 [2–99]	74 [4–99]	62 [2–97]	0.03
BM blasts at Dx (%)	74 [12–98]	74 [12–98]	73 [16–98]	0.23
FAB classification				
AML-M0	9 (3)	9 (5)	0	0.01
AML-M1	100 (32)	67 (35)	33 (27)	0.18
AML-M2	55 (17)	37 (19)	18 (15)	0.38
AML-M4	53 (17)	25 (13)	28 (23)	0.03
AML-M5	48 (15)	23 (12)	25 (20)	0.06
AML-M6	2 (1)	1 (0.5)	1 (0.8)	1.00
AML-M7	1 (0.3)	1 (0.5)	0	1.00
Not classifiable	48 (15)	30 (16)	18 (15)	0.95
Mutations — n (%)				
*NPM1*	186 (59)	119 (62)	67 (54)	0.25
*FLT3-*ITD	130 (41)	63 (33)	67 (54)	< 0.01
*FLT3*-TKD	19 (6)	11 (6)	8 (7)	0.96
*DNMT3A*	131 (41)	78 (40)	53 (43)	0.72
bZIP in-frame *CEBPA* ^†^	17 (5)	15 (8)	2 (2)	0.04
*RUNX1*	41 (13)	18 (9)	23 (19)	0.02
*ASXL1*	23 (7)	10 (5)	13 (11)	0.12
*TP53*	1 (0.3)	1 (0.5)	0	1.00
*IDH1*	41 (13)	32 (17)	9 (7)	0.03
*IDH2*	47 (15)	38 (20)	9 (7)	< 0.01
*IDH2* R140	37 (12)	30 (16)	7 (6)	0.01
*IDH2* R172	10 (3)	8 (4)	2 (2)	0.36
*NRAS*	43 (14)	32 (17)	11 (9)	0.08
*KRAS*	14 (4)	9 (5)	5 (4)	1.00
*SRSF2*	24 (8)	16 (8)	8 (7)	0.71
*ZRSR2*	4 (1)	1 (1)	3 (2)	0.33
*SF3B1*	10 (3)	5 (3)	5 (4)	0.69
*STAG2*	17 (5)	11 (6)	6 (5)	0.95
*TET2*	59 (19)	33 (17)	26 (21)	0.45
*EZH2*	9 (3)	4 (2)	5 (4)	0.49
*BCOR*	13 (4)	10 (4)	3 (4)	0.36
*U2AF1*	6 (2)	2 (1)	4 (3)	0.32
*PTPN11*	36 (11)	21 (11)	15 (12)	0.86
*WT1*	33 (10)	15 (8)	18 (15)	0.08
Mutational subgroups				
*NPM1* mut/*FLT3-*ITD pos/*DNMT3A* mut	59 (19)	29 (15)	30 (24)	0.05
*FLT3*-ITD neg/bZIP *CEBPA* neg ^†^	172 (54)	118 (61)	54 (44)	< 0.01
*NPM1* mut/*FLT3*-ITD neg/bZIP *CEBPA* neg	91 (29)	69 (36)	22 (18)	< 0.01
*NPM1* WT/*FLT3*-ITD neg/bZIP *CEBPA* neg	81 (26)	49 (25)	32 (26)	1.00
MRGM	92 (29)	49 (25)	43 (35)	0.09
ELN 2022 classification				< 0.01
Favorable	108 (34)	84 (44)	24 (20)	
Intermediate	128 (41)	69 (36)	59 (48)	
Adverse	80 (25)	40 (21)	40 (33)	
CR rate — n (%)	255 (81)	164 (85)	91 (74)	0.02
HSCT in CR1 — n (%)	66 (21)	40 (21)	26 (21)	1.00

NB. Percentage may not add up to 100% because of rounding. * WBC count at diagnosis was missing for 2 patients. † Three patients with bZIP in-frame *CEBPA* mutation also had co-occurring *FLT3*-ITD mutation. Therefore, the subset of patients without both *FLT3*-ITD and bZIP in-frame *CEBPA* includes 172 patients. *Dx* diagnosis, *FAB* French-American-British classification, *neg* negative, *pos* positive

### Next-generation sequencing

Whole transcriptome sequencing was performed using the Illumina HiSeq 2000 or NovaSeq 6000 system with RNA extracted from mononuclear cells of AML patient diagnostic samples. Gene expression was normalized in transcripts per million (TPM), then log-transformed and standardized into Z-scores [22]. Variant calling for identification of gene mutations was performed using RNA sequencing data as previously described [23] and confirmed with exome sequencing data*.* Single-cell RNA sequencing data was studied in 22 patient samples (Additional file 1: Supplementary methods).

### Identification of candidate gene expression biomarkers

We evaluated associations between the continuous expression of each protein-coding or long non-protein coding genes (*n* = 27,740) and OS or relapse-free survival (RFS) using Cox proportional hazards (CPH) regression models (Additional file 1: Fig. S1). We included age, white blood cell count and *NPM1*, *FLT3*-ITD, *DNMT3A*, bZIP *CEBPA*, *ASXL1* and *RUNX1* mutations as covariables in multivariable analyses (MVA). To identify predictive gene expression markers associated with benefit from HSCT, we evaluated interaction terms between gene expression and HSCT as a time-dependent (TD) variable (HSCT-TD) in CPH models for OS and RFS [24]. To identify the optimal cutoff of our candidate gene expression marker, we used different methods including the Youden index on receiver operating characteristic (ROC) curves and analysis of the prognostic and predictive impacts for all possible cutoffs of gene expression (Additional file 1: Supplementary methods, Fig. S2-S3).

### Statistical analyses

We analyzed associations between gene expression and CR rates, OS, RFS and cumulative incidence of relapse (CIR) with standard endpoint definitions reported by ELN 2022 [4]. OS and RFS were calculated using the Kaplan–Meier method and differences between groups were tested with the log-rank test. Hazard ratios (HRs) were calculated using CPH models with 95% confidence intervals (CI). The Fine and Gray method was used to calculate CIR with death in remission considered as a competing risk to relapse. All survival times were censored at time of HSCT in CR1 unless stated otherwise, to avoid the confounding effect of HSCT in survival analyses*.* The impact of HSCT in CR1 was evaluated by testing interaction terms between covariables and HSCT-TD [24]. We performed a 6-month landmark analysis to illustrate the benefit from HSCT in CR1 according to candidate gene expression marker. *P *values < 0.05 were considered statistically significant. Statistical analyses were performed using R version 4.0.2.

## Results

### Identification of *IL1R1* expression as a prognostic and predictive biomarker

We identified *IL1R1* as the top gene significantly associated with OS and RFS in univariable and multivariable analyses and with significant interaction with HSCT-TD (*p* < 0.05) (Additional file 1: Fig. S1). Using the Youden index, the optimal cutoff for dichotomization of *IL1R1* expression was 2.0 TPM, corresponding to a specificity of 82.5% and a sensitivity of 49.4% to predict 3-year OS (Additional file 1: Fig. S2). Testing for all possible cutoffs of *IL1R1* expression, cutoffs between the 35th and 98th percentiles (0.90 to 25.75 TPM) were statistically significant in univariable and multivariable analyses for OS and RFS (Additional file 1: Fig. S2). HSCT in CR1 was beneficial in patients with high expression of *IL1R1* with all the cutoffs evaluated between the 10th and 90th percentiles (Additional file 1: Fig. S3). For this manuscript, the cutoff of *IL1R1* expression was set at 2.0 TPM (61st percentile) to optimize both its prognostic and predictive impacts. *IL1R1* expression was slightly higher in patients who had a bone marrow sample sequenced versus those who had a peripheral blood sample sequenced (median BM 1.96 TPM vs PB 0.89 TPM, *p* < 0.01, Additional file 1: Fig. S4). However, this difference was only observed in patients without myelomonocytic differentiation (median BM 1.72 TPM vs PB 0.82 TPM, *p* < 0.01), but not in patients with myelomonocytic differentiation (median BM 2.05 TPM vs PB 2.02 TPM). There was no difference in *IL1R1* expression between the two sequencing cohorts Leucegene 2 and 3 (median, 1.43 TPM vs 1.49 TPM, respectively, *p* = 0.86).

### Patient characteristics according to *IL1R1* expression

The characteristics of the Leucegene IRC AML cohort are presented in Table 1. With a cutoff at 2.0 TPM, 123 (39%) patients had high expression of *IL1R1* (*IL1R1*^high^) and 193 (61%) patients had low expression of *IL1R1* (*IL1R1*^low^). Patients with *IL1R1*^high^ were older (median age difference 5 years, *p* < 0.01) with a trend for higher WBC at diagnosis. A greater proportion of patients with *IL1R1*^high^ had AML with myelomonocytic or monocytic differentiation (43% vs 25% for *IL1R1*^low^; *p* < 0.01). As expected, *NPM1* (59%), *FLT3*-ITD (41%) and *DNMT3A* (41%) mutations were frequently detected. *FLT3-*ITD and *RUNX1* mutations were more frequent in patients with *IL1R1*^high^ whereas *IDH1, IDH2* R140 and bZIP *CEBPA* mutations were more frequent in patients with *IL1R1*^low^. The frequency of *NPM1* mutations was similar between groups. There was a trend for a higher frequency of any of the MRGM among patients with *IL1R1*^high^ (35% vs 25% for *IL1R1*^low^, *p* = 0.09). Treatments received by patients are detailed in Additional file 1: Table S1.

### *IL1R1* expression is an independent predictor of clinical outcomes in IRC AML

**Fig. 1:**
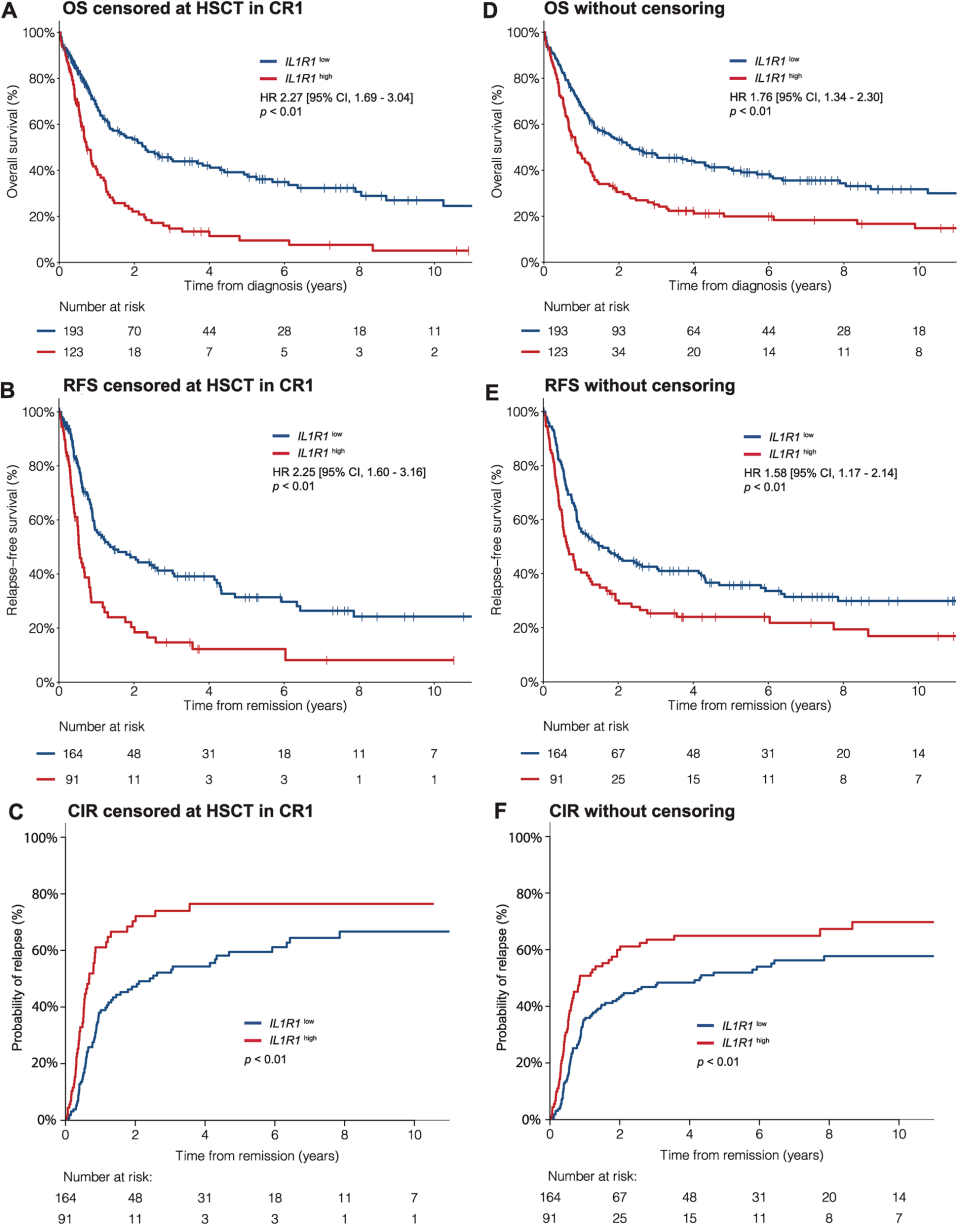
Clinical outcomes according to *IL1R1* expression in the Leucegene IRC AML cohort. **A** OS **B** RFS and **C** CIR for patients with *IL1R1*^low^ (blue line) and *IL1R1*^high^ (red line) with survival times censored at time of HSCT in CR1. **D** OS **E** RFS and **F** CIR for patients according to *IL1R1* expression without censoring at time of HSCT in CR1. *IL1R1* expression is dichotomized on the 61st percentile corresponding to 2.0 TPM

After induction chemotherapy, 255/316 (81%) patients achieved CR. The CR rate was 74% in patients with *IL1R1*^high^ versus 85% in patients with *IL1R1*^low^ (Odds ratio [OR] 1.99, *p* = 0.02). This association was independent from age, the only other variable associated with CR (adjusted OR 1.86,* p* = 0.03). With a median follow-up of 7.2 years, the median OS and 5-year OS rates were 9.0 months and 10% in patients with *IL1R1*^high^ versus 27.6 months and 38% in patients with *IL1R1*^low^ (HR 2.27, *p* < 0.01, Fig. 1A).

Among patients who achieved CR, the median RFS and 5-year RFS rates were 6.4 months and 12% versus 16.4 months and 31% in patients with *IL1R1*^high^ and *IL1R1*^low^, respectively (HR 2.25, *p* < 0.01, Fig. 1B). The 5-year CIR rate was 17% higher in patients with *IL1R1*^high^ (76%, *IL1R1*^high^ vs 59%, *IL1R1*^low^, *p* < 0.01, Fig. 1C), but there was no difference in cumulative incidence of death (CID) between groups (Additional file 1: Fig. S5). When times were not censored at time of HSCT, *IL1R1*^high^ remained significantly associated with worse outcomes, but the differences between groups were of lesser magnitude (Fig. 1D-F). Additional details of clinical outcomes according to *IL1R1* expression are available in Additional file 1: Table S2. In MVA, *IL1R1*^high^ was independently associated with both OS (HR 1.78, *p* < 0.01) and RFS (HR 1.77, *p* < 0.01) (Table 2, Additional file 1: Table S3). *IL1R1* expression was significantly associated with OS and RFS regardless of the tissue sample sequenced (BM vs PB) and the sequencing cohort (Leucegene 2 vs 3) (Additional file 1: Fig. S6).

**Table 2:** Multivariable analyses for OS and RFS including *IL1R1* expression

Characteristic	OS		RFS	
	HR [95% CI]	*p* value	HR [95% CI]	*p* value
Age ≥ 60 years old	2.20 [1.60–3.03]	< 0.01	1.79 [1.23–2.59]	< 0.01
WBC ≥ 50 × 10^9^/L	1.24 [0.90–1.71]	0.18	1.30 [0.90–1.88]	0.16
*NPM1* mutation	0.66 [0.45–0.96]	0.03	0.47 [0.30–0.75]	< 0.01
*FLT3-*ITD mutation	2.44 [1.73–3.44]	< 0.01	2.03 [1.37–3.02]	< 0.01
bZIP *CEBPA* mutation	0.44 [0.13–1.44]	0.17	0.24 [0.06–1.01]	0.05
*RUNX1* mutation	0.98 [0.65–1.48]	0.92	1.00 [0.61–1.65]	0.99
*ASXL1* mutation	1.22 [0.71–2.08]	0.48	1.53 [0.79–2.96]	0.21
*DNMT3A* mutation	1.41 [1.03–1.94]	0.03	2.01 [1.34–3.00]	< 0.01
*IL1R1*^high^	1.78 [1.29–2.45]	< 0.01	1.77 [1.22–2.56]	< 0.01

Analysis with censoring at time of HSCT in CR1. Similar analyses without censoring at time of HSCT in CR1 are available in the Additional file 1: Table S3. High expression of *IL1R1* above 2.0 TPM

**Fig. 2:**
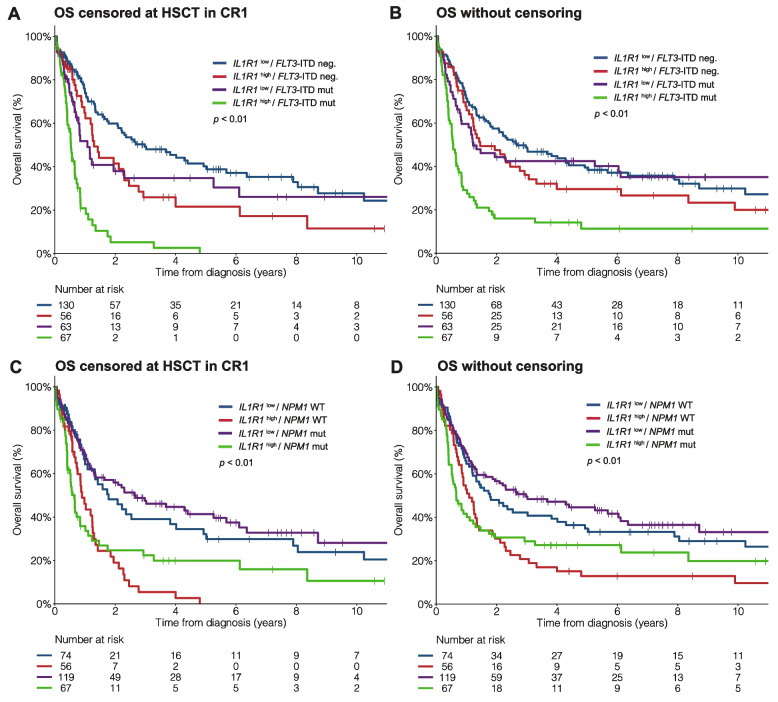
Clinical outcomes according to *IL1R1* expression and *NPM1* or *FLT3*-ITD mutations in the Leucegene IRC AML cohort. OS according to *IL1R1* expression and *FLT3*-ITD mutation status (**A**) with censoring at time of HSCT in CR1 and (**B**) without censoring at time of HSCT in CR1. OS according to *IL1R1* expression and *NPM1* mutation status (**C**) with censoring at time of HSCT in CR1 and (**D**) without censoring at time of HSCT in CR1

 Importantly, although *IL1R1*^high^ was associated with *FLT3*-ITD mutation (*FLT3*-ITD +), *IL1R1* expression had a significant prognostic impact both in patients with and without *FLT3*-ITD mutation (Fig. 2A-B, Additional file 1: Fig. S7)

In patients without *FLT3*-ITD, the 3-year OS rate was 26% versus 49% in patients with *IL1R1*^high^ and *IL1R1*^low^, respectively. In patients with *FLT3*-ITD +, the 3-year OS rate was 5% versus 35% in patients with *IL1R1*^high^ and *IL1R1*^low^, respectively. Similarly, *IL1R1*^high^ had a significant prognostic impact both in patients with and without *NPM1* mutation (Fig. 2C-D, Additional file 1: Fig. S7) and in both younger (< 60 years) and older (≥ 60 years) patients (Additional file 1: Fig. S8). *IL1R1* expression improved the prognostic stratification in patients with intermediate or adverse ELN 2022 risk, but not in patients with favorable risk (Additional file 1: Fig. S9). However, when using a higher cutoff for *IL1R1* expression (75th percentile, 3.0 TPM), patients with *IL1R1*^high^ had lower OS among those with favorable ELN 2022 risk (HR 2.24, *p* = 0.04) (Additional file 1: Fig. S10).

### Expression of *IL1R1* predicts the benefit from HSCT in CR1 in IRC AML

**Fig. 3:**
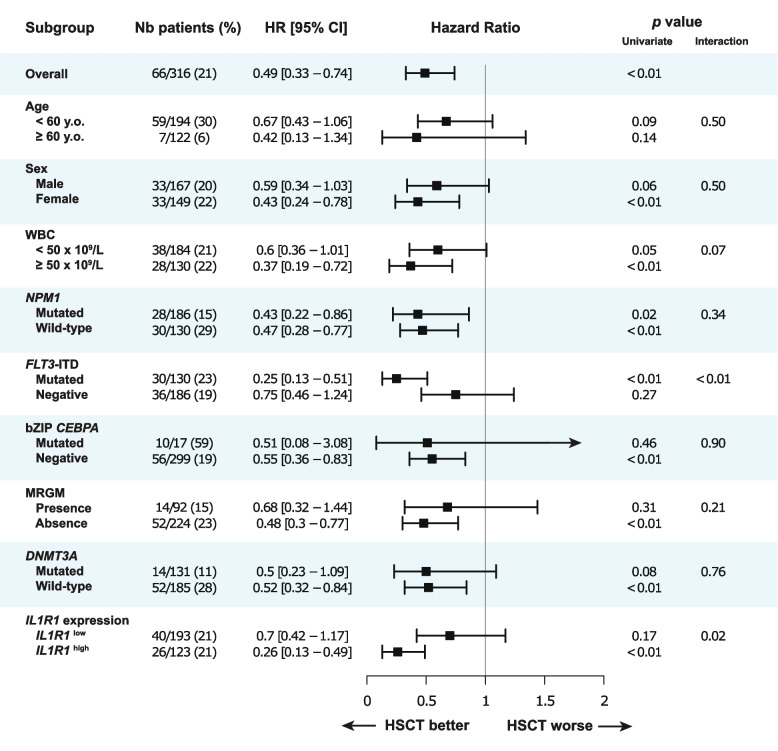
Forest-plot for the benefit of HSCT in CR1 within various clinicopathological subgroups of patients. The endpoint used for these analyses was OS. HR and *p *values were obtained using HSCT in CR1 as a time-dependent (TD) variable in CPH models. Interaction terms were tested between HSCT-TD and covariables in CPH models. The number and proportion of patients who have undergone HSCT in CR1 is represented for each group in the second column

In our cohort, 66/316 (21%) patients have undergone HSCT in CR1 and those patients were younger and had lower frequencies of *NPM1* and *DNMT3A* mutations (Additional file 1: Table S4). Among transplanted patients, the median age was 50 years (range, 21–64), which can be explained by the fact that 65 years was the maximal age for eligibility to HSCT in Quebec between 2001 and 2018. Interestingly, the frequency of *FLT3*-ITD mutation is similar between patients who proceeded to HSCT or not (45% vs 40%) mostly because *FLT3* mutational testing became widely available around 2010 in Canada and the *FLT3*-ITD mutation status was unknown for many patients included in the Leucegene cohort to inform decision regarding transplant. In patients who proceeded to HSCT in CR1, the median time from diagnosis to transplant was 4.8 months (range, 1.9–10.3), with no difference between patients with *IL1R1*^low^ (4.9 months, range 1.9–10.1) and patients with *IL1R1*^high^ (4.6 months, range 2.1–10.3) (*p* = 0.27). The transplant rate was also similar in both groups (21% [40/193] in *IL1R1*^high^ versus 21% [26/123] in *IL1R1*^low^, *p* = 1.00) (Additional file 1: Table S1). In the Leucegene cohort of patients with *de novo* IRC AML, HSCT in CR1 improved both OS (HR 0.49, *p* < 0.01) and RFS (HR 0.57, *p* < 0.01) (Fig. 3, Additional file 1: Fig. S11).

**Fig. 4:**
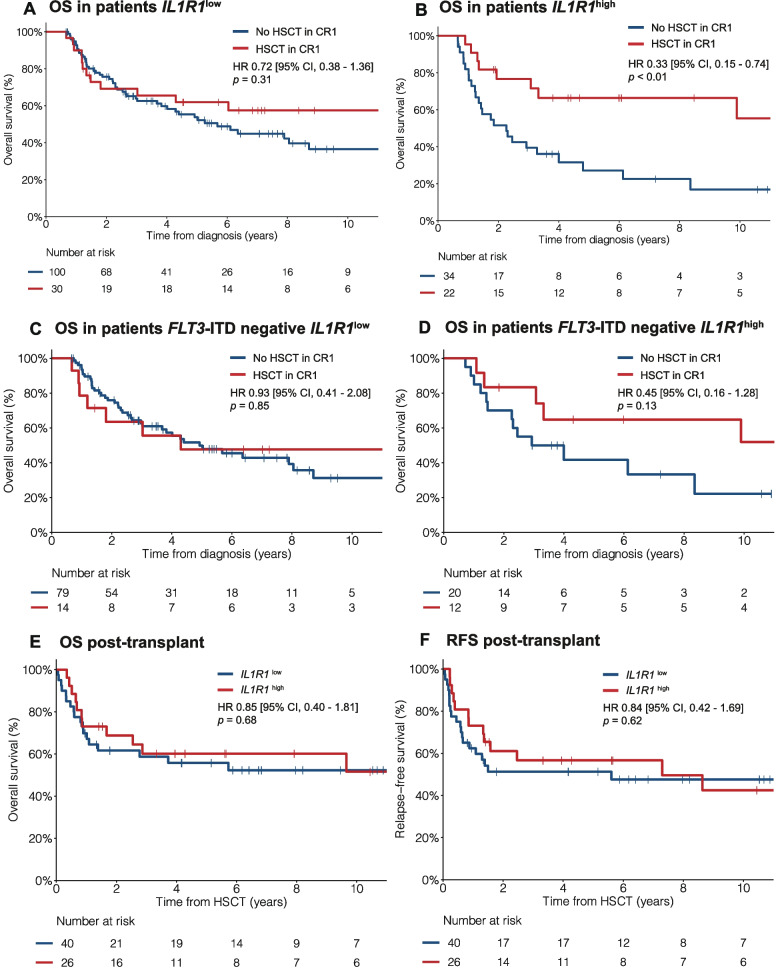
Impact of HSCT in CR1 on survival outcomes according to *IL1R1* expression. **A-D** Landmark analysis comparing survival outcomes in patients who proceeded to HSCT in CR1 (red line) versus those who did not (blue line). Landmark time was established at 6 months. **A** OS according to HSCT in CR1 in patients with *IL1R1*^low^. **B** OS according to HSCT in CR1 in patients with *IL1R1*^high^. **C** OS according to HSCT in CR1 in patients *FLT3*-ITD negative and *IL1R1*^low^. **D** OS according to HSCT in CR1 in patients *FLT3*-ITD negative and *IL1R1*^high^. **E** OS and **F** RFS post-HSCT in patients with *IL1R1*^low^ (blue line) and *IL1R1*^high^ (red line) among those who have undergone HSCT in CR1. Survival times were calculated from the date of HSCT in CR1 (panels **E–F**)

Expression of *IL1R1* modified the impact of HSCT in CR1 with a significant interaction between HSCT-TD and *IL1R1* expression as a continuous variable (OS, *p* < 0.01; RFS, *p* < 0.01) and binary variable (OS, *p* = 0.02; RFS, *p* = 0.02). HSCT in CR1 significantly improved OS and RFS in patients with *IL1R1*^high^ (HR for OS 0.26, *p* < 0.01; HR for RFS 0.32, *p* < 0.01), but not significantly in patients with *IL1R1*^low^ (HR for OS 0.70, *p* = 0.17; HR for RFS 0.75, *p* = 0.25) (Fig. 3, Additional file 1: Fig. S11). With a 6-month landmark analysis, the 5-year OS rates were 62% versus 54% (HR 0.72, *p* = 0.31) for patients with *IL1R1*^low^ and 67% versus 27% (HR 0.33, *p* < 0.01) for patients with *IL1R1*^high^ in patients who underwent HSCT or not, respectively (Fig. 4A-B).

**Fig. 5:**
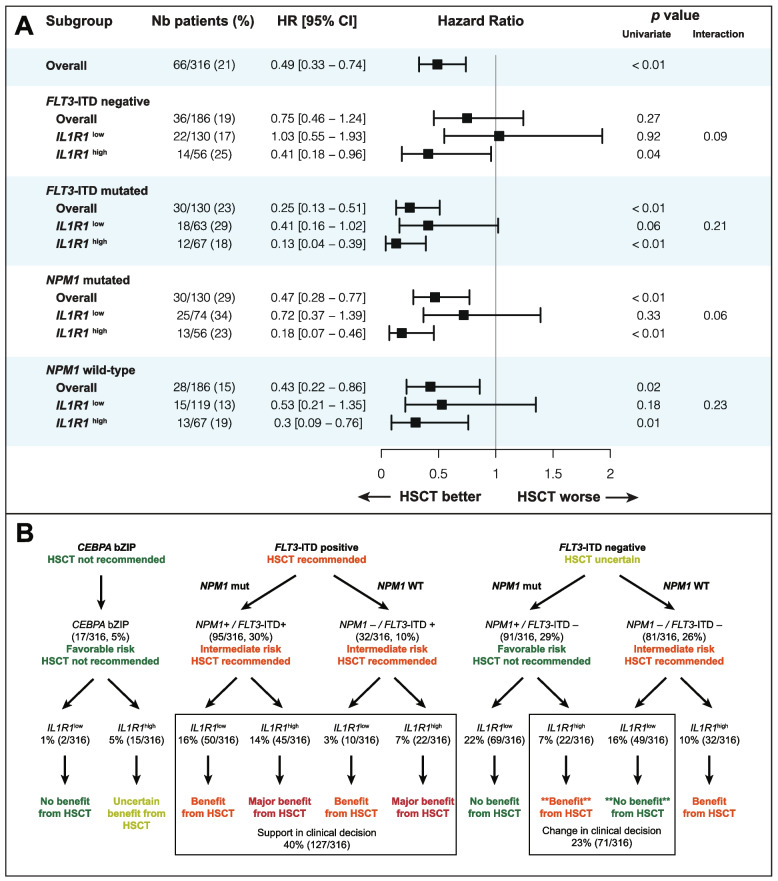
Benefit from HSCT in CR1 and clinical utility of *IL1R1* expression according to mutational subgroups. **A** Forest plot for the benefit from HSCT in CR1 on OS in the Leucegene IRC AML cohort according to *IL1R1* expression in relation to *FLT3*-ITD and *NPM1* mutational status. HR and *p *values were obtained using HSCT in CR1 as a time-dependent (TD) variable in CPH models. Interaction terms were tested between HSCT-TD and covariables in CPH models. The number and proportion of patients who have undergone HSCT in CR1 is represented for each group in the second column. **B** Clinical utility of *IL1R1* expression to guide the decision for HSCT in CR1 according to mutational subgroups

With a 6-month landmark analysis among patients without *FLT3*-ITD mutation, the 5-year OS rates were 48% versus 50% (HR 0.93, *p* = 0.85) for patients with *IL1R1*^low^ (*n* = 93) and 65% versus 42% (HR 0.45, *p* = 0.13) for patients with *IL1R1*^high^ (*n* = 32) in patients who underwent HSCT or not, respectively (Fig. 4C-D). Among patients without *FLT3*-ITD mutation and specifically negative for *NPM1* mutation, HSCT in CR1 was not beneficial in patients with *IL1R1*^low^ (HR 0.94, 95% CI 0.36–2.43, *p* = 0.90) whereas HSCT in CR1 significantly improved survival in those with *IL1R1*^high^ (HR 0.26, 95% CI 0.07–0.92, *p* = 0.04) (Additional file 1: Fig. S12). Among patients without *FLT3*-ITD, but with *NPM1* mutation, the 4 patients with *IL1R1*^high^ who proceeded with HSCT in CR1 remained alive in CR whereas 13 patients with *IL1R1*^high^ who didn’t proceed with HSCT in CR1 had a 5-year OS of 69% [95% CI, 48–99%] (Additional file 1: Fig. S12). Apart from *IL1R1* expression, significant interaction with HSCT-TD was observed for *FLT3*-ITD mutations confirming the known benefit of HSCT in these patients. The benefit from HSCT in CR1 was greater among patients with *FLT3*-ITD + (HR for OS 0.25, *p* < 0.01) than in patients without *FLT3*-ITD mutation (HR for OS 0.75, *p* = 0.27) (Fig. 3). Importantly, when testing the impact of HSCT as a time-dependent variable among patients without *FLT3*-ITD mutation, patients with *IL1R1*^high^ benefited from HSCT (HR for OS 0.41, *p* = 0.04) whereas patients with *IL1R1*^low^ had no benefit from HSCT (HR for OS 1.03, *p* = 0.92) (Fig. 5A).

Among patients with *FLT3*-ITD +, all patients benefited from HSCT irrespective of *IL1R1* expression, although the benefit was only marginally significant in patients with *IL1R1*^low^ (HR for OS 0.41, *p* = 0.06). Among patients with *FLT3*-ITD + and *IL1R1*^high^, 6/12 (50%) patients who underwent HSCT were alive in CR1 at last follow-up compared to 1/35 (3%) patients who did not undergo HSCT (HR for OS 0.13, *p* < 0.01). Among patients who have undergone HSCT in CR1, *IL1R1*^high^ was no longer associated with OS post-transplant (HR 0.85, *p* = 0.68) and RFS (HR 0.84, *p* = 0.62) indicating that HSCT abrogated the adverse impact of *IL1R1*^high^ (Fig. 4E-F). After HSCT, the 5-year OS and RFS rates were 56% and 51%, respectively, in patients with *IL1R1*^low^ compared to 60% and 57%, respectively, in patients with *IL1R1*^high^ (Fig. 4E-F, Additional file 1: Table S5). In patients with *IL1R1*^high^, the 5-year OS post-transplant was higher compared to 5-year OS censored at time of HSCT in CR1 for patients within each of the ELN 2022 risk categories (Additional file 1: Fig. S9).

### Clinical utility of *IL1R1* expression

*IL1R1* expression improved the prognostic stratification of patients with IRC AML with ELN 2022 intermediate and adverse risks (Additional file 1: Fig. S9). Overall, using a multivariable CPH model for OS including the ELN 2022 risk classification and *IL1R1* expression as covariables, the concordance index (c-statistic) improved to 0.661 compared to 0.639 when only the 2022 ELN risk classification was included (absolute difference 0.022) (Additional file 1: Table S6). Most importantly, *IL1R1* expression would support the decision for HSCT in 172/316 (54%) patients without bZIP *CEBPA* or *FLT3*-ITD mutations in whom the indication is most uncertain (Fig. 5B). Considering current recommendations for HSCT in CR1 in patients with IRC AML, clinical decision regarding HSCT in CR1 could be modified in 71/316 (23%) patients with IRC AML when using *IL1R1* expression (Fig. 5B). Twenty-two patients (7% of total cohort) with *NPM1* mutations without *FLT3*-ITD mutation who would not normally undergo HSCT in CR1 would be best managed by proceeding to HSCT because of *IL1R1*^high^*.* Additionally, 49 patients (16% of total cohort) without *NPM1*, *FLT3*-ITD and bZIP *CEBPA* mutation who would normally undergo HSCT in CR1 would be best managed by consolidation chemotherapy instead of HSCT because of *IL1R1*^low^. In patients with *FLT3*-ITD + (40%, 127/316), *IL1R1* expression may differentiate patients with dismal outcomes who will inevitably relapse without HSCT (*IL1R1*^high^) from patients with a more nuanced risk–benefit ratio (*IL1R1*^low^) therefore supporting the decision to proceed with HSCT.

To facilitate the clinical evaluation of *IL1R1* expression as a novel biomarker, we developed and validated a RT-qPCR test for measurements of *IL1R1* expression using 260 diagnostic specimens from the Leucegene *de novo* IRC AML cohort (Additional file 1: Supplementary methods and Table S7-S8). The resulting quantifications were highly correlated with the existing RNA sequencing data for the same samples (*r* = 0.90, *p* < 2.2e-16) (Additional file 1: Fig. S13). Using ROC curve analyses and similar methods for identification of the cutoff by RNA-sequencing, the cutoff for positivity of the test (high *IL1R1* expression value) was set at 1354 normalized copy number (NCN), at the 61st percentile of the *IL1R1* NCN distribution (Additional file 1: Fig. S13). The prognostic impact of high expression of *IL1R1* measured by RT-qPCR was validated clinically in the Leucegene cohort and was statistically significant for both OS and RFS (HR for OS 1.87, 95% CI 1.37–2.55, *p* < 0.01; HR for RFS 1.94, 95% CI 1.34–2.80, *p* < 0.01) (Additional file 1: Fig. S13). We also confirmed that *IL1R1* expression measured by RT-qPCR predicts the benefit from HSCT in CR1. In patients with *IL1R1*^high^ (≥ 1354 NCN), HSCT-TD significantly improved OS (HR 0.18, *p* < 0.01), but not in patients with *IL1R1*^low^ (< 1354 NCN) (HR 0.63, *p* = 0.10).

### Bulk and single-cell transcriptomic associations with *IL1R1* expression

**Fig. 6:**
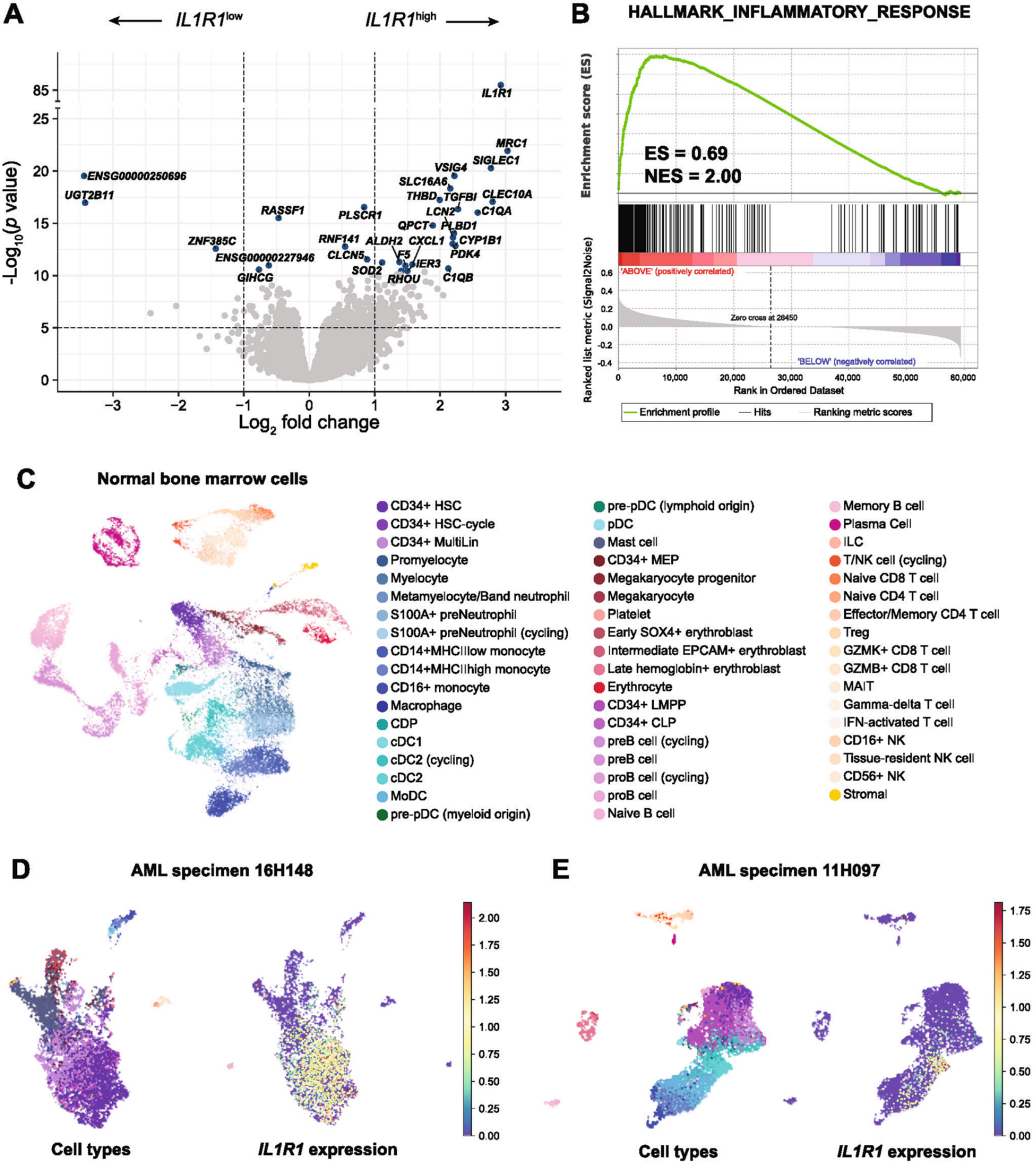
Bulk and single-cell transcriptomic associations with *IL1R1* expression. **A** Volcano plot representing differential gene expression analyses between patient samples with low and high expression of *IL1R1*. Analyses were performed using the DESeq2 method [44]. Genes overexpressed in samples with *IL1R1*^low^ appear on the left and genes overexpressed in samples with *IL1R1*^high^ appear on the right. The names of the top 30 genes are displayed. **B** Gene Set Enrichment Analysis between patient samples with low and high expression of *IL1R1* using the Broad Institute of Massachusetts Institute of Technology method [45]. There is a significant enrichment of inflammatory response gene sets in patients with high expression of *IL1R1*. **C** UMAP representation after Harmony integration of normal bone marrow single-cell RNA sequencing data with each cell being color coded according to their cell type as predicted by the classifier. **D** UMAP representation of single-cell RNA sequencing data for AML sample 16H148 with cell type predictions (left panel) and single-cell expression of *IL1R1* (right panel). In this sample, *IL1R1* is predominantly expressed in immature CD34-positive blasts. **E** UMAP representation of single-cell RNA sequencing data for sample 11H097 with cell type predictions (left panel) and single-cell expression of *IL1R1* (right panel). In this sample, *IL1R1* is mostly expressed in conventional dendritic cells 2 (cDC2)

To investigate why patients with *IL1R1*^high^ may benefit from HSCT, we performed differential gene expression analysis between patients with *IL1R1*^low^ versus *IL1R1*^high^ (Fig. 6A). The top gene overexpressed in patients with *IL1R1*^high^ was *MRC1* which encodes for the mannose receptor present at the cell surface of macrophages and immature dendritic cells [25, 26]. Other genes associated with monocytic, macrophages or dendritic cells differentiation were significantly overexpressed in patients with *IL1R1*^high^ (including *SIGLEC1, CLEC10A, TGFBI, C1QA, C1QB* highlighted in Fig. 6A; and *CD14*, CD163*, CD300E, MSR1* in the top 50 overexpressed genes).

When conducting gene set enrichment analysis, inflammatory response was in the top 5 of hallmark gene sets enriched in patients with *IL1R1*^high^, along with coagulation, epithelial-mesenchymal transition, complement and angiogenesis hallmarks (Fig. 6B, Additional file 1: Fig. S14). In order to understand which cells specifically express *IL1R1*, we first analyzed *IL1R1* expression values in bulk transcriptomic data obtained from normal blood and bone marrow populations sequenced in the Leucegene project. These results confirmed that *IL1R1* is expressed in these normal cell populations, especially granulocytes and monocytes (Additional file 1: Fig. S15). We then studied single-cell RNA sequencing of normal bone marrow from the Human cell atlas and from 22 AML patient samples from the Leucegene AML cohort (20 patient samples previously reported [27] and two new samples with known high *IL1R1* expression based on bulk transcriptomics). In normal bone marrow cells (Fig. 6C), *IL1R1* is expressed in CD14-positive monocytes, in conventional dendritic cells 2 (cDC2) and in stromal cells (Additional file 1: Fig. S16). In AML samples, *IL1R1* expression had two distinct patterns exemplified by specimens 16H148 and 11H097 which had higher *IL1R1* expression (Fig. 6D-E). In specimen 16H148, *IL1R1* was predominantly expressed in immature CD34-positive myeloblasts and was highly correlated at the single-cell level with genes associated with an immature phenotype such as *DNTT, SPINK2, AVP, CD34* and *FAM30A* (Fig. 6D, Additional file 1: Fig. S16). In contrast, in specimen 11H097, *IL1R1* was rather expressed in cDC2 and was associated with the expression of genes associated with these cells such as *MRC1*, *CLEC10A* and *CD1C* (Fig. 6E, Additional file 1: Fig. S16).

Finally, we also evaluated whether the adverse prognostic impact of *IL1R1* was shared by other genes involved in the IL1 signaling pathway. Among the 37 genes evaluated, only high expression of *IL1RAP* was significantly associated with adverse OS and RFS as previously reported by our group [28] (Additional file 1: Table S9). However, *IL1RAP* expression did not predict the benefit from HSCT in CR1.

## Discussion

In this report, we show that *IL1R1* gene expression in diagnostic patient samples with *de novo* IRC AML is associated with adverse prognosis and may identify patients who benefit from HSCT in CR1.

Because clinical trials with randomization of HSCT are challenging to conduct, the indication for HSCT in CR1 in patients with IRC AML is mostly supported by retrospective studies or genetic randomization studies [12, 14, 29, 30]. Meta-analyses of these studies have shown that overall, HSCT in CR1 appears beneficial in patients with IRC AML [8–11]. However, the only randomized trial ever performed to address this question, the ETAL-1 study, has shown that HSCT decreases CIR, but does not improve OS in these patients [15]. These conflicting data between previous non-randomized studies and the ETAL-1 study underscore the uncertainty about indication for HSCT in IRC AML and highlight the need for biomarkers to identify those who benefit from HSCT in CR1. To our knowledge, expression of *IL1R1* is the first gene expression prognostic marker specifically identified and developed to predict the benefit of HSCT in CR1. In patients with IRC AML without *FLT3*-ITD mutation, only patients with *IL1R1*^high^ appeared to benefit from HSCT in CR1 in our cohort. This finding is potentially relevant for patients with *NPM1* +/*FLT3*-ITD– who are generally not offered HSCT in CR1, although they still have a high rate of relapse of approximately 50% [6]. In patients with *FLT3*-ITD +, although HSCT in CR1 is beneficial irrespective of *IL1R1* expression, patients with *IL1R1*^high^ are at very high risk of relapse which may influence transplant-related decisions such as selection of donor, conditioning regimen and immunosuppression post-transplant to optimize outcomes. However, it is important to note that only 10/149 (7%) of patients with *FLT3* mutations have received midostaurin as part of their treatment in our cohort. The prognostic impact of *IL1R1* expression and its potential to predict the benefit from transplant remains uncertain in patients with *FLT3* mutations who receive midostaurin or other FLT3 inhibitors with intensive chemotherapy. In patients without *NPM1*, *FLT3*-ITD and bZIP *CEBPA* mutation, which represent a substantial proportion of patients with IRC AML (~ 25%), there is currently no well-established genetic marker to guide the decision for HSCT in CR1. Myelodysplasia-related gene mutations (MRGM), which are frequent in these patients, have been included in the adverse-risk category in the ELN 2022 classification based on its strong association with secondary AML and worse clinical outcomes [4, 31]. However, data on the benefit of HSCT specifically in patients harboring any of these mutations is lacking. Based on our data, both patients with and without MRGM benefited from HSCT. In our cohort, patients with *NPM1*–/*FLT3*-ITD– and with *IL1R1*^low^ did not benefit from transplant and would likely be better treated with consolidation chemotherapy without HSCT whereas those with *IL1R1*^high^ clearly benefited from HSCT. Therefore, in patients with *NPM1*–/*FLT3*-ITD–, *IL1R1* expression might be a better biomarker than MRGM to select patients who should proceed to HSCT. Although the data from our cohort is promising and interesting, the possible ability of *IL1R1* expression to predict the benefit from HSCT in CR1 remains to be validated in external independent cohorts, especially for subgroups with the greatest potential clinical utility such has AML with *NPM1* +/*FLT3*-ITD– or *NPM1*–/*FLT3*-ITD–. Unfortunately, we could not validate our findings regarding the prediction of benefit from HSCT in CR1 with *IL1R1* expression because of the limited size of publicly available cohorts of patients with IRC AML treated with intensive chemotherapy with gene expression data at diagnosis. Furthermore, to properly validate the possible ability of *IL1R1* expression to identify the patients who benefit from HSCT, detailed transplant-related clinical data, including transplant dates, are required to perform survival analyses with censoring at time of transplant and analysis with transplant as a time-dependent variable.

The adverse prognostic impact of *IL1R1*^high^ was previously reported in an independent external cohort of 70 patients with AML although the association with benefit of HSCT was not studied [32]. In this paper, the significant association of *IL1R1* expression with OS and EFS was also validated in the TCGA (*n* = 162) and TARGET (*n* = 254) AML cohorts [32]. Altogether, the prognostic impact of *IL1R1* expression has now been validated in four independent patient cohorts, including the Leucegene IRC AML cohort described in this report. We demonstrated that *IL1R1*^high^ is associated with adverse OS and RFS and is independent of age, WBC at diagnosis and ELN 2022 risk classification. Although we confirm with our cohort that *IL1R1* expression is strongly and independently associated with clinical outcomes, the clinical utility of a prognostic biomarker is quite limited without any evidence that it may predict response or benefit from specific therapies. Further research is warranted to better define the potential clinical utility of *IL1R1* expression as a biomarker in newly diagnosed IRC AML beyond its use to estimate prognosis.

Apart from gene mutations and cytogenetics abnormalities, persistence of measurable residual disease (MRD) after intensive chemotherapy is now a well-recognized adverse prognostic factor in AML and is increasingly utilized to identify patients who benefit from HSCT, especially in *NPM1*-mutated AML [33–35]. Unfortunately, patient samples included in this study were collected before 2019 at a time when MRD testing was not routinely performed in clinical practice for AML in Canada. Since molecular and flow cytometry MRD assessments are now widely implemented to support decisions regarding indication from HSCT in CR1, the lack of MRD data is a significant limitation of our study. Consequently, future studies are warranted to evaluate whether *IL1R1* expression is an independent prognostic factor from MRD status for prognosis assessment and identification of patients who benefit from HSCT in CR1. Our results showing that *IL1R1* is also expressed in normal blood and bone marrow populations indicate that *IL1R1*^high^ is a useful prognostic biomarker only at diagnosis and would not be useful for MRD monitoring. Nonetheless, irrespective of its association with MRD persistence, *IL1R1* expression has the advantage of being tested at diagnosis which allows to identify before starting induction therapy which patients will have suboptimal response to chemotherapy and may benefit from alternative treatment approaches and from HSCT in CR1. MRD testing also poses multiple technical challenges including the variability of results according to technologies utilized (flow cytometry vs molecular testing) and the requirement of significant resources and time [33]. With the aim of facilitating future studies with *IL1R1* expression and possible future clinical implementation, we developed a simple and inexpensive RT-qPCR test to measure *IL1R1* expression. Measurement of *IL1R1* expression by this RT-qPCR test is highly correlated with measurements with RNA-sequencing and we confirmed that *IL1R1* expression in NCN measured by RT-qPCR was associated with OS and RFS in our cohort. Before clinical implementation of this RT-qPCR test may be envisioned, clinical validation of the test in external independent cohorts and standardization of the quantification results across multiple clinical laboratories are important additional test development milestones yet to be performed for *IL1R1* expression. As part of test development, the cutoff that we identified and selected for *IL1R1* expression in our cohort would also need to be validated in external cohorts.

To generate hypotheses to understand why patients with high expression of *IL1R1* have poor clinical outcomes and may benefit from HSCT in CR1, we performed differential gene expression analyses, GSEA and single-cell RNA sequencing analyses. AML samples with *IL1R1*^high^ more frequently had monocytic differentiation and had enrichment in inflammatory hallmark gene sets*.* In differential gene expression analyses, *IL1R1*^high^ samples had increased expression of many genes associated with monocytic, macrophage and dendritic cells, such as *MRC1*, which was the top gene overexpressed in *IL1R1*^high^ samples. In our single-cell RNA sequencing analysis, *MRC1* expression was correlated with *IL1R1* expression only when the latter was expressed in cells of monocytic lineage including dendritic cells. In other AML samples, *IL1R1* was rather expressed in immature blastic leukemia cells suggesting that more than one mechanism may be at play. The increased expression of *IL1R1* in immature blastic cells and its correlation with leukemia stem cell (LSC) markers which have been included in LSC gene expression prognostic signatures (*CD34, SPINK2, FAM30A*) [36, 37], suggest that some cell intrinsic factors may also be involved in the poor prognosis associated with *IL1R1*^high^. IL1 has been demonstrated to drive proliferation of leukemia cells by both an autocrine and paracrine manner [38]. However, when evaluating the prognostic impact of genes involved in the IL1 signaling pathway, only *IL1RAP* expression, the IL1 receptor accessory protein, was significantly associated with adverse outcomes, but it did not predict benefit from HSCT in CR1. It is unknown whether the adverse prognosis associated with *IL1R1*^high^ is associated with activation of the IL1 signaling pathways in leukemia cells or higher expression of *IL1R1* is a bystander of another process.

The identification of *IL1R1* as a biomarker in AML also suggests potential interest in targeting the IL1 pathway or other inflammatory pathways as novel therapeutic avenues in AML [39]. Competitive inhibitors (anakinra) or direct inhibitors (canakinumab) of IL1 have been successfully used in other settings such as prevention of cardiovascular disease, rheumatoid arthritis and auto-inflammatory syndromes and would likely merit study in AML [40–42]. Interestingly, small molecule inhibitors of the downstream signaling pathway of IL1 have shown to be effective in preclinical models of AML [38] and, more specifically, IRAK1/4 inhibitors are currently being evaluated in clinical studies in patients with AML or myelodysplastic syndromes [43].

## Conclusion

Our results demonstrate that *IL1R1* expression at diagnosis of AML is a promising new biomarker to improve prognostication in patients with IRC and may guide the decision to recommend HSCT or consolidation chemotherapy in these patients. External validation of *IL1R1* expression as a prognostic and predictive biomarker and further studies evaluating other pro-inflammatory markers might improve risk stratification and prediction of response to HSCT or other therapies in AML.

## Supplementary Information

Additional file 1. Supplementary methods. Table S1 Treatments received by patients in the Leucegene cohort. Table S2 Clinical outcomes according to *IL1R1* expression. Table S3 Multivariable analyses for OS and RFS without censoring at time of HSCT in CR1. Table S4 Characteristics of patients according to HSCT status. Table S5 Clinical outcomes post-HSCT according to *IL1R1* expression. Table S6 C-index in MVA including ELN 2022 and *IL1R1* expression as covariable. Table S7 Primers and probes for the *IL1R1* RT-qPCR test. Table S8 Analytical validation performance specifications for the *IL1R1* RT-qPCR test. Table S9 Prognostic analyses of genes involved in the IL1 signaling pathway and other related genes. Fig. S1 Identification of *IL1R1* expression as a prognostic and predictive biomarker. Fig. S2 Identification of the optimal cutoff value for dichotomization of *IL1R1* expression. Fig. S3 Benefit from HSCT in CR1 for overall survival using different cutoffs for *IL1R1* expression. Fig. S4 Expression of *IL1R1* according to the type of sample sequenced and myelomonocytic differentiation of the AML. Fig. S5 Cumulative incidence of death in remission according to *IL1R1* expression. Fig. S6 Prognostic impact of *IL1R1* expression according to the type of sample sequenced and the sequencing cohort. Fig. S7 Prognostic impact of *IL1R1* expression according to *NPM1* or *FLT3*-ITD mutational status. Fig. S8 Prognostic impact of *IL1R1* expression according to age. Fig. S9 Prognostic impact of *IL1R1* expression according to 2022 ELN risk classification. Fig S10 Prognostic impact of *IL1R1* with a higher cutoff value in patients with ELN 2022 favorable-risk AML. Fig. S11 Benefit from HSCT in CR1 for RFS in clinicopathological subgroups of patients. Fig. S12 Impact of HSCT in CR1 on survival outcomes according to *NPM1* mutational status and *IL1R1* expression in patients *FLT3*-ITD negative. Fig. S13 Correlation between *IL1R1* expression quantification by the RT-qPCR test and RNA sequencing and clinical validation of the *IL1R1* RT-qPCR test in the Leucegene cohort. Fig. S14 Additional Gene Set Enrichment Analyses between patients with high and low expression of *IL1R1*. Fig. S15 Analysis of *IL1R1* expression in normal blood and bone marrow populations and in acute leukemias sequenced in the Leucegene project. Fig. S16 Single-cell RNA sequencing of normal bone marrow and AML specimens.

Additional file 2. Table S10 Clinical data.

## Data Availability

The laboratory data for the Leucegene AML cohort used in this manuscript are detailed in the Additional file 2: Table S10 (Excel file). The RNA sequences for the 316 patient samples analyzed in this study are available in the Gene Expression Omnibus repository (GEO accession number GSE232130). Single-cell RNA sequencing dataset is available under GEO accession number GSE241989. Non-identifiable clinical data for the Leucegene AML prognostic cohort are available to academic investigators with research ethics committee approval in accordance with the procedures of the Quebec Leukemia Cell Bank (bclq.org).

## Abbreviations

AML: Acute myeloid leukemia
BM: Bone marrow
CI: Confidence interval
CID: Cumulative incidence of death
CIR: Cumulative incidence of relapse
CPH: Cox proportional hazards
CR1: First complete remission
Dx: Diagnosis
ELN: European LeukemiaNet
FAB: French-American-British classification
HR: Hazard ratio
HSCT: Hematopoietic stem cell transplantation
IRC: Intermediate-risk cytogenetics
MRGM: Myelodysplasia-related gene mutations
MVA: Multivariable analysis
mut: Mutated
OR: Odds ratio
OS: Overall survival
PB: Peripheral blood
ROC: Receiver operating characteristic
RFS: Relapse-free survival
TD: time-dependent
TPM: Transcripts per million
WBC: White blood cell count
WT: Wild type
