# Supplementary material for: High IL1R1 expression predicts poor survival and benefit from stem cell transplant in intermediate-risk acute myeloid leukemia from the Leucegene cohort
Source: Biomark Res. 2025 Oct 23;13:133. doi: 10.1186/s40364-025-00827-6 (PMC12548205; doi:10.1186/s40364-025-00827-6)
Supplement: Supplementary file 1 — Additional file 1. Supplementary methods. Table S1 Treatments received by patients in the Leucegene cohort. Table S2 Clinical outcomes according to IL1R1 expression. Table S3 Multivariable analyses for OS and RFS without censoring at time of HSCT in CR1. Table S4 Characteristics of patients according to HSCT status. Table S5 Clinical outcomes post-HSCT according to IL1R1 expression. Table S6 C-index in MVA including ELN 2022 and IL1R1 expression as covariable. Table S7 Primers and probes for the IL1R1 RT-qPCR test. Table S8 Analytical validation performance specifications for the IL1R1 RT-qPCR test. Table S9 Prognostic analyses of genes involved in the IL1 signaling pathway and other related genes. Fig. S1 Identification of IL1R1 expression as a prognostic and predictive biomarker. Fig. S2 Identification of the optimal cutoff value for dichotomization of IL1R1 expression. Fig. S3 Benefit from HSCT in CR1 for overall survival using different cutoffs for IL1R1 expression. Fig. S4 Expression of IL1R1 according to the type of sample sequenced and myelomonocytic differentiation of the AML. Fig. S5 Cumulative incidence of death in remission according to IL1R1 expression. Fig. S6 Prognostic impact of IL1R1 expression according to the type of sample sequenced and the sequencing cohort. Fig. S7 Prognostic impact of IL1R1 expression according to NPM1 or FLT3-ITD mutational status. Fig. S8 Prognostic impact of IL1R1 expression according to age. Fig. S9 Prognostic impact of IL1R1 expression according to 2022 ELN risk classification. Fig S10 Prognostic impact of IL1R1 with a higher cutoff value in patients with ELN 2022 favorable-risk AML. Fig. S11 Benefit from HSCT in CR1 for RFS in clinicopathological subgroups of patients. Fig. S12 Impact of HSCT in CR1 on survival outcomes according to NPM1 mutational status and IL1R1 expression in patients FLT3-ITD negative. Fig. S13 Correlation between IL1R1 expression quantification by the RT-qPCR test and RNA sequencing and clinical vali [file 40364_2025_827_MOESM1_ESM.pdf]

Supplementary Information

High *IL1R1* expression predicts poor survival and benefit from stem cell transplant in intermediate-risk acute myeloid leukemia from the Leucegene cohort

Guillaume Richard-Carpentier[1,2*], François Béliveau[3], Sandrine Lacoste[3], Banafsheh Khakipoor[4], Véronique Lisi[4], Michael Vladovsky[4], Miriam Marquis[3], Jean-François Spinella[5], Patrick Gendron[5], Sébastien Lemieux[5,6], Vincent-Philippe Lavallée[4,5,7], Guy Sauvageau[3,5,8,9], Josée Hébert[3,5,8,9*]

[1] Princess Margaret Cancer Centre, University Health Network, Toronto, ON, Canada.
[2] Department of Medicine, Division of Medical Oncology and Hematology, Temerty Faculty of Medicine, University of Toronto, Toronto, ON, Canada.
[3] Quebec Leukemia Cell Bank, Centre de recherche de l'Hôpital Maisonneuve-Rosemont, Montréal, QC, Canada.
[4] Centre Hospitalier Universitaire Sainte-Justine, Montréal, QC, Canada.
[5] Institute for Research in Immunology and Cancer, Université de Montréal, Montréal, QC, Canada.
[6] Department of Computer Science and Operations Research, Université de Montréal, Montréal, QC, Canada.
[7] Department of Pediatrics, Faculty of Medicine, Université de Montréal, Montréal, QC, Canada.
[8] Division of Hematology-Oncology and Cell Therapy, Hôpital Maisonneuve-Rosemont, Montréal, QC, Canada.
[9] Department of Medicine, Faculty of Medicine, Université de Montréal, Montréal, QC, Canada.

Short Title: *IL1R1* expression and benefit from HSCT in AML

Keywords: Acute Myeloid Leukemia, Transcriptomics, Gene expression, Predictive Biomarker, Prognosis, Allogeneic Stem Cell Transplantation

***Corresponding authors:**

Guillaume Richard-Carpentier, MD FRCPC
Princess Margaret Cancer Centre, University Health Network
University of Toronto, Department of Medicine, Division of Medical Oncology and Hematology
610 University Avenue, OPG Building, 700 U, Toronto, Ontario, Canada, M5G 2M9
E-mail: guillaume.richard-carpentier@uhn.ca

Josée Hébert, MD FRCPC
Division of Hematology-Oncology and Cell Therapy, Hôpital Maisonneuve-Rosemont
Université de Montréal, Department of Medicine, Faculty of Medicine
5415 Boulevard l'Assomption, Montréal, QC, Canada, H1T 2M4
E-mail: josee.hebert@umontreal.ca

Table of Contents

1. Supplementary methods .... - 3 -

1.1 Sample processing and next-generation sequencing. .... - 3 -
1.2 Mutation identification and validation. .... - 3 -
1.3 RNA sequencing data processing for identification of gene expresson biomarkers. .... - 4 -
1.4 Identification of the optimal cutoff for *IL1R1* expression. . ... - 4 -
1.5 Additional statistical methods and survival endpoints. .... - 6 -
1.6 Development and validation of a RT-qPCR test for *IL1R1* expression. .... - 6 -
1.7 Single-cell RNA sequencing. . ... - 9 -

2. Supplementary Tables .... - 10 -

Table S1. Treatments received by patients in the Leucegene cohort. .... - 10 -
Table S2 Clinical outcomes according to *IL1R1* expression. .... - 11 -
Table S3 Multivariable analyses for OS and RFS without censoring at time of HSCT in CR1. .... - 12 -
Table S4 Characteristics of patients according to HSCT status. .... - 13 -
Table S5 Clinical outcomes post-HSCT according to *IL1R1* expression. .... - 14 -
Table S6 C-index in MVA including ELN 2022 and *IL1R1* expression as covariable. .... - 15 -
Table S7 Primers and probes for the *IL1R1* RT-qPCR test. .... - 16 -
Table S8 Analytical validation performance specifications for the *IL1R1* RT-qPCR test. .... - 17 -
Table S9 Prognostic analyses of genes involved in the IL1 signaling pathway and other related genes. - 18 -

3. Supplementary Figures .... - 19 -

Fig. S1 Identification of *IL1R1* expression as a prognostic and predictive biomarker. .... - 19 -
Fig. S2 Identification of the optimal cutoff value for dichotomization of *IL1R1* expression. .... - 20 -
Fig. S3 Benefit from HSCT in CR1 for OS using different cutoffs for *IL1R1* expression. .... - 22 -
Fig. S4 Expression of *IL1R1* according to the type of sample sequenced and myelomonocytic differentiation of the AML..- 23 -
Fig. S5 Cumulative incidence of death in remission according to *IL1R1* expression. .... - 24 -
Fig. S6 Prognostic impact of *IL1R1* expression according to the type of sample sequenced and the sequencing cohort..- 25 -
Fig S7 Prognostic impact of *IL1R1* expression according to *NPM1* or *FLT3*-ITD mutational status. .... - 26 -
Fig. S8 Prognostic impact of *IL1R1* expression according to age. .... - 27 -
Fig. S9 Prognostic impact of *IL1R1* expression according to 2022 ELN risk classification. .... - 28 -
Fig. S10 Prognostic impact of *IL1R1* with a higher cutoff value in patients with ELN 2022 favorable-risk AML. .... - 29 -
Fig.S11 Benefit from HSCT in CR1 for RFS in clinicopathological subgroups of patients. .... - 30 -
Fig. S12 Impact of HSCT in CR1 on survival outcomes according to *NPM1* mutational status and *IL1R1* expression in patients *FLT3*-ITD negative..- 31-
Fig. S13 Correlation between *IL1R1* expression quantification by the RT-qPCR test and RNA sequencing and clinical validation of the *IL1R1* RT-qPCR test in the Leucegene cohort....................................- 32-
Fig. S14 Additional Gene Set Enrichment Analyses between patients with high and low expression of *IL1R1*. .... - 34 -
Fig. S15 Analysis of *IL1R1* expression in normal blood and bone marrow populations and in acute leukemias sequenced in the Leucegene project..- 35-
Fig. S16 Single-cell RNA sequencing of normal bone marrow and AML specimens .... - 36 -

4. References for Supplementary Information .... - 37 -

1. Supplementary methods

1.1 Sample processing and next-generation sequencing

Samples included in the Leucegene cohort were collected with an informed consent from patients treated in 8 adult hospitals in Quebec, Canada between 2001 and 2019 by the Quebec Leukemia Cell Bank, a biobank certified by the Canadian Tissue Repository Network (https://www.ctrnet.ca/en/home/). The Quebec Leukemia Cell Bank program is approved annually by the Research Ethics Board of Hôpital Maisonneuve-Rosemont. Mononuclear cells were prepared from bone marrow or blood samples using Ficoll and cryopreserved at -80°C in TRIzol reagent or in nitrogen liquid (10% DMSO). RNA was extracted using TRIzol reagent according to the manufacturer's instruction (Invitrogen / Life Technologies) with additional purification on RNeasy mini columns (Qiagen). Libraries were constructed according to TruSeq Protocol (Illumina) and sequencing was performed using an Illumina HiSeq 2000 or NovaSeq 6000 sequencing system. Pseudo-alignment and quantification of transcripts were done using Kallisto (v0.46.0) with RNA sequences from Gencode version 32 as reference [1]. The Tximport R package was used to obtain quantification per gene [1]. Samples from the Leucegene cohort of intensively treated patients with *de novo* acute myeloid leukemia (AML) and intermediate-risk cytogenetics (IRC) (316 samples) were sequenced in two different sequencing cohorts: 163 samples in 2014 (Leucegene 2) and 153 samples in 2018 - 2019 (Leucegene 3). Whole transcriptome sequencing data from the two cohorts have been reanalyzed together with the methods described above to provide uniform data.

1.2 Mutation identification and validation

Variants in the Leucegene 2 cohort were identified as previously reported [2]. Variants for the Leucegene 2 and 3 cohorts were detected from RNA-sequencing data using FreeBayes version 1.3.1 from aligned data (STAR version 2.7.1a) and from exome sequencing using Mutect2 version 4.1.3.0. Variants outside of the coding regions were excluded, and only nonsynonymous variants (SNP or Indel) were considered. Variants identified in normal controls (most often representing polymorphisms) were filtered out. Known single nucleotide polymorphisms (SNP) (dbSNP, version 137) were also removed, except for those in known

leukemia “hotspots”. Mutations with a variant allelic frequency (VAF) ≥ 5% and ≥ 8 variant reads were included, with lower VAFs tolerated for mutational hotspots as previously reported by our group. Because *NRAS* and *KRAS* mutations are commonly found in minor clones, a VAF of ≥ 3% was accepted if ≥ 15 variant reads were present in “hotspots” of those genes (*N/KRAS* G12, G13 and Q61). The variants were classified according to the American College of Medical Genetics and Genomics (ACMG) variant classification and only “pathogenic” or “likely pathogenic” variants were considered as gene mutations. The VarSome tool (versions 11.1.4 to 11.7.7) was used for this classification (https://varsome.com/).

Results from PCR with capillary electrophoresis-based DNA fragment analysis (*FLT3*-ITD) and targeted next-generation sequencing for detection of *NPM1*, *FLT3*, *CEBPA* and other mutations from local certified clinical laboratories were used to validate variants if available and were accorded priority in case of discrepancy. *FLT3*-ITD mutations were also studied with a k-mer approach, as described previously [3].

1.3 RNA sequencing data processing for identification of gene expression biomarkers

Gene expression levels were normalized and reported as transcripts per million (TPM). We studied protein-coding genes and long non-coding RNAs, excluding all genes with minimal expression for which all patient samples had an expression below 1.0 TPM. For gene expression to be used in statistical models, we performed a logarithmic transformation of base 10 after adding 1 to gene expression levels to avoid zero values. The gene expression data was then standardized into Z-scores (mean-centered at 0 with variance of 1) to have pseudo-normal distributions and limit mean-variance correlation and respect assumptions in logistic and Cox regression models [4]. RNA sequencing data for the complete Leucegene cohort (691 samples) are available in the Gene Expression Omnibus (GEO) repository (accession number GSE232130).

1.4 Identification of the optimal cutoff for *IL1R1* expression

To facilitate potential clinical implementation for risk stratification and decision-making for indication of hematopoietic stem cell transplantation (HSCT) in first complete remission (CR1), we dichotomized the expression of *IL1R1* by dividing patients into two groups with either high ($IL1R1^{high}$) or low ($IL1R1^{low}$) expression. To select the best cutoff to optimize the

prognostic and predictive impact of *IL1R1* expression, we used three different complementary approaches. First, we performed receiver operating characteristic (ROC) curves representing sensitivity and specificity of *IL1R1* expression at all possible cutoffs to predict 3-year overall survival (OS) or relapse-free survival (RFS) (**Fig. S2**, only shown for OS). Using the Youden index, the optimal cutoff was identified at 2.0 TPM, corresponding to a specificity of 82.5% and a sensitivity of 49.4% to predict 3-year OS. Second, we calculated hazard ratios (HR) and *p*-values for dichotomized *IL1R1* expression in univariable analysis (UVA) and multivariable analysis (MVA) for RFS and OS with all possible cutoffs in our cohort which corresponds to the total number of patients minus one (n = 315) (**Fig. S2**). Cutoffs between the 35th and 98th percentiles of *IL1R1* expression (0.90 to 25.75 TPM) were associated with significant *p*-values ($p < 0.05$) in Cox proportional hazards (CPH) models for OS and RFS in UVA and MVA. With increasing cutoff values for *IL1R1* expression, the HRs for OS and RFS also increased, but included a gradually smaller proportion of patients in the *IL1R1*^{high} group. Third, we evaluated the impact of HSCT in CR1 on OS and RFS in patients with high expression of *IL1R1* using 9 different cutoffs (from 10th percentile to 90th percentile) (**Fig. S3**). The HSCT in CR1 was considered as a time-dependent (HSCT-TD) variable in extended CPH models and interaction terms between HSCT-TD and dichotomized *IL1R1* expression were calculated. With increasing cutoff values of *IL1R1* expression, the benefit of HSCT in CR1 in patients with high *IL1R1* expression consistently increased (corresponding to lower HR for the benefit of HSCT in CR1 with higher cutoffs of *IL1R1* expression) (**Fig. S3**). HSCT in CR1 was beneficial in patients with high expression of *IL1R1* at all the cutoffs evaluated, whereas HSCT in CR1 was not beneficial in patients with low expression of *IL1R1*, except when the cutoffs were set at the 80th and 90th percentiles (i.e. when most of the IRC AML cohort was classified as *IL1R1*^{low}). Overall, cutoffs between the 50th and 75th percentiles were optimal to identify patients who benefit the most from HSCT in CR1 while including the largest proportion of patients possible. With the results obtained from these three different methods, we set the cutoff at 2.0 TPM which corresponds to the 61st percentile of *IL1R1* expression. This cutoff of *IL1R1* expression was optimal for both the prognostic impact and prediction of benefit from HSCT in CR1 in patients with IRC AML.

1.5 Additional statistical methods and survival endpoints

Associations between *IL1R1* expression groups and patients' clinicopathological characteristics were tested with Wilcoxon rank sum test for continuous variables and with Chi-squared or Fisher's exact test for categorical variables. Associations between clinicopathological and complete remission (CR) rates were tested with logistic regression models. Overall survival (OS) was defined as the time from diagnosis to death from any cause or last follow-up. Relapse-free survival (RFS) was defined as the time from CR to relapse, death from any cause or last follow-up, whichever came first. Cumulative incidence of relapse (CIR) was defined as the time from CR to relapse with considering death in remission as a competing risk with the Fine and Gray method. Cumulative incidence of death (CID) in remission was defined as the time from CR to death with considering relapse as a competing risk with the Fine and Gray method. In patients who have undergone HSCT in CR1, OS and RFS post-transplant were evaluated using the transplant date as start time.

1.6 Development and validation of a RT-qPCR test for *IL1R1* expression

<u>RNA purification</u>

Total RNA was extracted from mononuclear cells of patient samples (bone marrow and/or peripheral blood) cryopreserved at -80°C in TRIzol (Invitrogen) since diagnosis. After addition of chloroform, mixing and centrifugation as recommended from the TRIzol™ manufacturer's protocol, the aqueous phase was mixed with 1 volume of isopropanol before loading on a column of the RNeasy Mini Kit (Qiagen). All subsequent steps of RNA purification were performed as recommended. RNA samples were quantified and checked for purity (ratio A260/A280) using Nanodrop 2000. Their integrity was verified using Bioanalyzer (Agilent Technologies) and only RNAs with RNA integrity number ≥ 7.0 were included in the study. RNA samples were stored at -80°C until the next step.

<u>Development and validation of the *IL1R1* RT-qPCR test</u>

Reverse transcriptions (RT) of 1 µg of RNA per sample were performed in a volume of 20 µl using the Readyscript cDNA synthesis Mix (Sigma) and then diluted with DNAse & RNAse free H_2O to a final volume of 50 µl. For each batch of RT reactions, a no template RT negative

control and the RNAs of HL-60 (high *IL1R1* expression) and OCI-AML2 (low *IL1R1* expression) cell lines were included as controls.

MIQE guidelines were followed in the design and validation of the *IL1R1* assay [5]. The amplicon overlaps the junction between exons 3 & 4 of the most prevalent *IL1R1* transcript (ENST00000410023.6), a junction that is present in 99.2% of the *IL1R1* transcripts identified in the RNA sequencing data of the Leucegene AML cohort. Primers and probe for *ABL1* (internal control) have been published previously by the Europe Against Cancer program [6].

For the assay, *IL1R1* and *ABL1* were amplified and quantified separately using plasmid-based standard curves prepared as described previously [7]. The quantitative PCR were carried out with a QuantStudio™ S7 Flex system (Applied Biosystems) using 5 µl (10%) of cDNA per well (or 5 µl of plasmid standard containing 10^6, 10^5, 10^4, 10^3, 10^2 or 10^1 copies) in a 25 µl final volume (1x TaqMan™ Fast Advance Master Mix, 300 nM of each primer, 200 nM FAM/ZEN/3' IBFQ probe (IDT)) (sequences in **Table S7**). Amplification was performed in 0.1 ml MicroAmp Fast 96-well plates (Applied Biosystems) in Fast mode with the following conditions: 2min at 50°C, 2min at 95°C, 50 cycles of 1s at 95°C followed by 20s at 60°C. All samples (patient samples, controls, standard curves) were analyzed in duplicates. For the analysis with the QuantiStudio Real-Time PCR software, the threshold was set to 0.1 and the average copy number (CN) for *IL1R1* and *ABL1* over the 2 duplicates was automatically calculated by the software based on the plasmid standard curves performed on the same plate. Finally, the normalized copy number (NCN) was calculated from this data: NCN = CN_{IL1R1} / CN_{ABL1} * 10000. For statistical analysis and graphic representation, NCNs were further log transformed as $\log_{10}$(NCN*1000+1).

An analytical validation adapted from the Clinical and Laboratory Standards Institute was also performed (CLSI documents EP05-A3, EP09-A3, EP06-A, EP17-A2, EP28-A3) for the assay (**Table S8**) [8-12].

Identification of the optimal cutoff for *IL1R1* expression measured by RT-qPCR

Following similar methods than for the identification of the optimal cutoff for *IL1R1* expression measured by RNA-sequencing described above, we aimed to identify and validate an optimal cutoff for *IL1R1* expression measured by RT-qPCR. First, using the correlation analysis between RNA-sequencing and RT-qPCR measurements for *IL1R1* expression, we determined approximative equivalent values of *IL1R1* expression in normalized copy number (NCN) corresponding to 2.0 TPM measured by RNA-sequencing (**Fig. S13A**). Depending on the use of non-transformed or log-transformed *IL1R1* expression values, an *IL1R1* expression value of 2.0 TPM by RNA-seq was equivalent to 1141 to 1502 NCN. The 61st percentile of *IL1R1* expression measured by RT-qPCR was 1354 NCN. Using ROC curve with all possible cutoffs of *IL1R1* expression by RT-qPCR, the cutoff of 1354 NCN was associated with a specificity of 74.3% and sensitivity of 44.2% to predict 3-year OS (**Fig. S13B**). We also evaluated all possible cutoffs of *IL1R1* expression by RT-qPCR in association with OS and RFS in univariable and multivariable analyses. In UVA for OS, *IL1R1* NCN cutoffs between the 28th and 100th percentiles were statistically significant with HRs ranging from 1.39 to 7.28 (**Fig. S13C**). In MVA for OS, *IL1R1* NCN cutoffs between the 56th and 99th percentiles were statistically significant with HRs ranging from 1.40 to 3.42 (data not shown). In UVA for RFS, *IL1R1* NCN cutoffs between the 34th and 100th percentiles were statistically significant with HRs ranging from 1.47 to 7.39 (**Fig. S13D**). In MVA for RFS, *IL1R1* NCN cutoffs between the 57th and 98th percentiles were statistically significant with HRs ranging from 1.50 to 4.59 (data not shown). Altogether, *IL1R1* NCN cutoffs between the 57th and 98th percentiles were significant in all analyses and we selected a cutoff at 1354 NCN (61st percentile) since it was the average of equivalent NCN values for 2.0 TPM measured by RNA-Seq with optimized specificity and sensitivity on ROC curves analyses.

Clinical validation of the cutoff for *IL1R1* expression measured by RT-qPCR

To confirm the clinical value and prognostic impact of *IL1R1* expression measured by RT-qPCR and validate the cutoff of 1354 NCN, we evaluated OS and RFS censored at time of HSCT in CR1 in patients with *IL1R1*^{low} (< 1354 NCN) and *IL1R1*^{high} (≥ 1354 NCN). Patients

with *IL1R1*^{high} by RT-qPCR had a statistically worse OS and RFS (**Fig. S13E-F**). We also confirmed that *IL1R1* expression measured by RT-qPCR was able to predict the benefit of HSCT in CR1 by testing the impact of HSCT as a time-dependent (HSCT-TD) variable in patients with *IL1R1*^{low} (< 1354 NCN) versus *IL1R1*^{high} (≥ 1354 NCN) and testing the interaction between HSCT-TD and *IL1R1* expression measured by RT-qPCR. In patients with *IL1R1*^{low} (< 1354 NCN), HSCT-TD did not significantly improve OS (HR 0.63, *p*=0.10) whereas it significantly improved OS in patients with *IL1R1*^{high} (≥ 1354 NCN) (HR 0.18, *p*<0.01) with a significant interaction between HSCT-TD and *IL1R1* expression measured by RT-qPCR (p=0.02). Results were similar for RFS with significant interaction between HSCT-TD and *IL1R1* expression measured by RT-qPCR (p=0.04).

1.7 Single-cell RNA sequencing

Single-cell RNA sequencing data was obtained and analyzed as previously described [13]. In brief, cryopreserved cells obtained from the Quebec Leukemia Cell Bank were thawed and washed before encapsulation in a Chromium Controller (10x Genomics) following the manufacturer’s protocol. The 3′ Next-GEM kit v3.1 was used, targeting the recovery of at least 6000 cells per sample. Libraries were prepared using single index adapters and sequenced on a NovaSeq 6000, with a target of 200 million reads per sample. FASTQ files were processed using Cell Ranger v5. Data filtering, normalization, and embedding were performed as previously described [13]. Cell annotation was conducted using ANNCast (https://github.com/lavalleelab/AMLclassifier).

2. Supplementary Tables

Table S1 Treatments received by patients in the Leucegene cohort.

Characteristic	Total cohort (n = 316)	*IL1R1*^{low} (n = 193)	*IL1R1*^{high} (n = 123)	*p* value
Type of induction chemotherapy				0.67
7+3 regimen (daunorubicin)	180 (57)	103 (53)	77 (63)	
7+3 regimen (idarubicin)	94 (30)	64 (33)	30 (24)	
7+3 regimen + GO	5 (2)	3 (2)	2 (2)	
7+3 regimen + midostaurin*	10 (3)	6 (3)	4 (3)	
HDAC + anthracycline	8 (3)	5 (3)	3 (2)	
HDAC + etoposide	3 (1)	1 (1)	2 (2)	
FLAG or FLAG-Ida	2 (1)	1 (1)	1 (1)	
ICE	14 (4)	10 (5)	4 (3)	
Number of inductions				0.17
1	234 (74)	148 (77)	86 (70)	
2	65 (21)	38 (20)	27 (22)	
3	16 (5)	6 (3)	10 (8)	
4	1 (0.3)	1 (1)	0	
Consolidation received**	219/254 (86)	144/163 (88)	75/91 (82)	0.26
Number of consolidations**				< 0.01
0	35/254 (14)	19/163 (12)	16/91 (18)	
1	43/254 (17)	22/163 (13)	21/91 (23)	
2	49/254 (19)	32/163 (20)	17/91 (19)	
3	61/254 (24)	36/163 (22)	25/91 (27)	
4	66/254 (26)	54/163 (33)	12/91 (13)	
HSCT in CR1***	66 (21)	40 (21)	26 (21)	1.00
Type of donor				0.62
MSD	45 (68)	26 (65)	19 (73)	
MUD	20 (30)	13 (32)	7 (27)	
Haploidentical	1 (2)	1 (2)	0	

* 10/149 (7%) of patients with *FLT3* mutations (130 with *FLT3*-ITD and 19 with *FLT3*-TKD) received midostaurin. ** A total of 255 patients achieved CR: 164/193 (85%) patients with *IL1R1*^{low} and 91/123 (74%) patients with *IL1R1*^{high}. One patient with *IL1R1*^{low} was lost to follow-up after achieving CR and the number of consolidation courses received, if any, is unknown for this patient. "Consolidation received" refers to the number of patients who received at least one cycle of consolidation chemotherapy. The 7+3 regimen received by the majority of patients consists of 7 days of continuous infusion of cytarabine 100-200 mg/m^2 per day and 3 days of bolus infusion of anthracycline, either daunorubicin (60-90 mg/m^2 per day) or idarubicin (12 mg/m^2 per day). *** 27/316 (8.5%) patients in CR2 and 3/316 (0.01%) patients in refractory state also received an allo-HSCT in this cohort. Abbreviations: GO, gemtuzumab ozogamicin; HDAC, high-dose cytarabine; FLAG, fludarabine, Ara-C, G-CSF; FLAG-Ida, fludarabine, Ara-C, G-CSF, idarubicin; ICE, idarubicin, cytarabine, etoposide; HSCT, allogeneic hematopoietic stem cell transplantation; CR1, first complete remission; MSD, matched sibling donor; MUD, matched unrelated donor.

Table S2 Clinical outcomes according to *IL1R1* expression.

Clinical outcome	Global cohort (n = 316)	*IL1R1*^{low} (n = 193)	*IL1R1*^{high} (n = 123)	*p* value
**CR rate – n (%)**	255 (81)	164 (85)	91 (74)	0.02
**Early death rate (< 30 days)**	17 (5)	10 (5)	7 (6)	1.00
**OS (w/o HSCT censoring)**				
Median – months [CI]	16.2 [14.3 – 24.9]	27.6 [19.8 – 51.6]	10.8 [8.1 – 14.9]	< 0.01
5-year rate – % [CI]	33 [27 – 38]	41 [34 – 49]	20 [14 – 29]	
**OS (with HSCT censoring)**				
Median – months [CI]	15.4 [12.4 – 21.4]	27.6 [17.1 – 51.4]	9.0 [7.7 – 12.3]	< 0.01
5-year rate – % [CI]	27 [22 – 34]	38 [31 – 48]	10 [5 – 20]	
**RFS (w/o HSCT censoring)**				
Median – months [CI]	12.7 [10.3 – 20.9]	19.0 [11.5 – 36.9]	7.8 [6.2 – 14.6]	< 0.01
5-year rate – % [CI]	31 [26 – 38]	36 [29 – 45]	24 [16 – 34]	
**RFS (with HSCT censoring)**				
Median – months [CI]	10.9 [9.8 – 15.7]	16.4 [11.3 – 36.9]	6.4 [5.9 – 9.7]	< 0.01
5-year rate – % [CI]	25 [19 – 33]	31 [23 – 42]	12 [6 – 25]	
**CIR (w/o HSCT censoring)**				
5-year rate – % [CI]	57 [50 – 63]	52 [44 – 60]	65 [55 – 75]	< 0.01
**CIR (with HSCT censoring)**				
5-year rate – % [CI]	65 [58 – 73]	59 [50 – 69]	76 [65 – 88]	< 0.01
**CID* (w/o HSCT censoring)**				
5-year rate – % [CI]	12 [8 – 16]	12 [7 – 18]	11 [5 – 18]	0.91
**CID* (with HSCT censoring)**				
5-year rate – % [CI]	10 [6 – 15]	9 [4 – 15]	11 [4 – 19]	0.23

* CID is defined as the time from remission to death in remission, with relapse considered as a competing event with the Fine and Gray method. CID applies only to patients who achieved complete remission. Abbreviations: CR, complete remission; OS, overall survival; w/o, without; HSCT, hematopoietic stem cell transplantation (allogeneic); RFS, relapse-free survival; CIR, cumulative incidence of relapse; CID, cumulative incidence of death (in remission).

Table S3 Multivariable analyses for OS and RFS without censoring at time of HSCT in CR1.

Characteristic	OS		RFS	
	**HR [95% CI]**	***p* value**	**HR [95% CI]**	***p* value**
Age ≥ 60 years old	2.20 [1.63 – 2.98]	< 0.01	1.71 [1.22 – 2.41]	< 0.01
WBC ≥ 50 x10^9/L	1.27 [0.95 – 1.69]	0.11	1.31 [0.95 – 1.81]	0.10
NPM1 mutation	0.66 [0.47 – 0.92]	0.01	0.48 [0.32 – 0.72]	< 0.01
FLT3-ITD mutation	1.79 [1.32 – 2.44]	< 0.01	1.48 [1.06 – 2.07]	0.02
bZIP *CEBPA* mutation	0.30 [0.12 – 0.76]	0.01	0.19 [0.07 – 0.54]	< 0.01
RUNX1 mutation	1.15 [0.79 – 1.67]	0.46	1.16 [0.75 – 1.79]	0.50
ASXL1 mutation	1.34 [0.81 – 2.21]	0.26	1.82 [0.98 – 3.38]	0.06
DNMT3A mutation	1.58 [1.17 – 2.13]	< 0.01	2.28 [1.57 – 3.32]	< 0.01
IL1R1^{high}	1.45 [1.09 – 1.92]	< 0.01	1.35 [0.99 – 1.86]	0.06

Analyses without censoring at time of HSCT in CR1. High expression of *IL1R1* above 2.0 TPM. Abbreviations: OS, overall survival; RFS, relapse-free survival; HSCT, allogeneic hematopoietic stem cell transplantation; CR1, first complete remission; WBC, white blood cells.

Table S4 Characteristics of patients according to HSCT status.

Characteristic	Total cohort (n = 316)	No HSCT in CR1 (n = 250)	HSCT in CR1 (n = 66)	*p* value
Age at Dx — median [range]	56 [20 – 78]	58 [20 – 78]	50 [21 - 64]	< 0.01
Age ≥ 60 years old	122 (39)	115 (46)	7 (11)	
Sex (male) — n (%)	167 (53)	134 (54)	33 (50)	0.70
WBC at Dx (x10^9/L)	37.0 [0.7 – 375.6]	36.3 [0.7 – 375.6]	38.0 [0.9 – 288.4]	0.43
WBC ≥ 50 x10^9/L*	130/314 (41)	102/248 (41)	28/66 (42)	0.96
PB blasts at Dx (%)	70 [2 – 99]	70 [2 – 99]	71 [8 - 98]	0.41
BM blasts at Dx (%)	74 [12 – 98]	73 [16 – 98]	78 [12 – 98]	0.53
FAB classification				0.03
AML-M0	9 (3)	9 (4)	0	
AML-M1	100 (32)	74 (30)	26 (39)	
AML-M2	55 (17)	41 (16)	14 (21)	
AML-M4	53 (17)	44 (18)	9 (14)	
AML-M5	48 (15)	39 (16)	9 (14)	
AML-M6	2 (1)	0	2 (3)	
AML-M7	1 (0.3)	1 (0.4)	0	
Not classifiable	48 (15)	42 (17)	6 (9)	
Mutations — n (%)				
NPM1	186 (59)	158 (63)	28 (42)	< 0.01
FLT3-ITD	130 (41)	100 (40)	30 (45)	0.51
FLT3-TKD	19 (6)	18 (7)	1 (2)	0.15
DNMT3A	131 (41)	117 (47)	14 (21)	< 0.01
bZIP in-frame *CEBPA* †	17 (5)	7 (3)	10 (15)	< 0.01
RUNX1	41 (13)	32 (13)	9 (14)	1.00
ASXL1	23 (7)	21 (8)	2 (3)	0.22
TP53	1 (0.3)	0	1 (2)	0.47
ELN 2022 classification				0.16
Favorable	108 (34)	95 (38)	33 (50)	
Intermediate	128 (41)	87 (35)	21 (32)	
Adverse	80 (25)	68 (27)	12 (18)	

NB. Percentage may not add up to 100% because of rounding. * WBC count at diagnosis was missing for 2 patients. † Ten of 17 patients (59%) with bZIP *CEBPA* mutation underwent HSCT in CR1. In many of these patients, the mutational status was unknown by clinicians since patients were diagnosed between 2001 and 2017, including a period before routine testing for *CEBPA* mutations. HSCT might have been indicated by high WBC count. Abbreviations: HSCT, allogeneic hematopoietic stem cell transplantation; CR1, first complete remission; Dx, diagnosis; WBC, white blood cell count; PB, peripheral blood; BM, bone marrow; FAB, French-American-British classification; ELN, European LeukemiaNet.

Table S5 Clinical outcomes post-HSCT according to *IL1R1* expression.

Clinical outcome †	Total patients (n = 66)	*IL1R1*^{low} (n = 40)	*IL1R1*^{high} (n = 26)	*p* value
**OS post-HSCT**				
Median – months [CI]	NR [34.4 – NR]	NR [16.7 – NR]	NR [30.5 – NR]	0.68
5-year rate – % [CI]	58 [47 – 71]	56 [42 – 74]	60 [44 – 83]	
**RFS post-HSCT**				
Median – months [CI]	87.6 [16.4 – NR]	67.3 [10.5 – NR]	87.6 [16.4 – NR]	0.62
5-year rate – % [CI]	53 [42 – 67]	51 [38 – 70]	57 [40 – 80]	
**CIR post-HSCT**				
5-year rate – % [CI]	31 [20 – 43]	28 [14 – 42]	36 [16 – 55]	0.48
**CID* post-HSCT**				
5-year rate – % [CI]	15 [7 – 24]	21 [8 – 34]	8 [0 – 18]	0.15

* CID is defined as the time from remission to death in remission, with relapse considered as a competing event with the Fine and Gray method. CID applies only to patients who achieved complete remission. † All survival times in this table are calculated from date of HSCT in CR1. Abbreviations: HSCT, allogeneic hematopoietic stem cell transplantation; OS, overall survival; RFS, relapse-free survival; CIR, cumulative incidence of relapse; CID, cumulative incidence of death (in remission).

Table S6 C-index in MVA including ELN 2022 and *IL1R1* expression as covariable.

CPH Model	OS §			RFS §		
	HR [95% CI]	*p* value	c-index	HR [95% CI]	*p* value	c-index
**Univariable analyses**						
ELN 2022			0.639			0.677
Favorable	0.30 [0.21 – 0.43]	< 0.01		0.28 [0.19 – 0.43]	< 0.01	
Adverse †	0.67 [0.48 – 0.94]	0.02		0.82 [0.55 – 1.22]	0.34	
IL1R1^{high}	2.27 [1.69 – 3.04]	< 0.01	0.593	2.24 [1.60 – 3.16]	< 0.01	0.603
**Multivariable analysis #1**						
ELN 2022			0.661			0.707
Favorable	0.31 [0.21 – 0.45]	< 0.01		0.28 [0.18 – 0.42]	< 0.01	
Adverse †	0.60 [0.42 – 0.84]	< 0.01		0.73 [0.49 – 1.09]	0.13	
IL1R1^{high}	2.15 [1.59 – 2.91]	< 0.01		2.15 [1.51 – 3.06]	< 0.01	
**Multivariable analysis #2**						
ELN 2022			0.700			0.728
Favorable	0.30 [0.21 – 0.45]	< 0.01		0.27 [0.18 – 0.42]	< 0.01	
Adverse †	0.58 [0.40 – 0.84]	< 0.01		0.66 [0.42 – 1.02]	0.06	
Age ≥ 60 years	2.19 [1.61 – 2.98]	< 0.01		2.08 [1.46 – 2.95]	< 0.01	
WBC ≥ 50 x 10^9/L	1.28 [0.93 – 1.75]	0.13		1.30 [0.91 – 1.87]	0.15	
IL1R1^{high}	1.97 [1.44 – 2.69]	< 0.01		1.98 [1.39 – 2.84]	< 0.01	

§ OS and RFS are censored at time of HSCT in CR1 for these analyses. † In our cohort of AML with intermediate-risk cytogenetics, patients with adverse ELN 2022 risk appear to have similar or slightly more favorable risk than patients with intermediate risk. This is attributable to the recategorization of *FLT3*-ITD into the ELN 2022 intermediate category whereas these patients had an adverse prognosis in our cohort (only few received midostaurin and a small proportion proceeded to HSCT in CR1). Also, MRGM are classified in the ELN 2022 adverse category, but in our cohort, presence of MRGM is not associated with an adverse prognosis among patients with non-mutated *NPM1* (i.e. wild-type) [14]. Abbreviations: C-index, concordance index; MVA, multivariable analysis; ELN, European LeukemiaNet; OS, overall survival; RFS, relapse-free survival; HR, hazard ratio; CPH, Cox proportional hazards models; WBC, white blood cell; HSCT, allogeneic hematopoietic stem cell transplantation; CR1, first complete remission; AML, acute myeloid leukemia; MRGM, myelodysplasia-related gene mutations.

Table S7 Primers and probes for the *IL1R1* RT-qPCR test.

Gene name (NCBI Gene ID)		Sequence (5'-3')	EAC code[6]
IL1R1 (3554)	F primer	TGGAGGCTGATAAATGCAAGGA	
	R primer	GGGTTAAGAGGACAGGGACG	
	Probe FAM/ZEN/IABkFQ	AGTGTCATCTGCAAATGAAATTGATGT	
ABL1 (25)	F primer	TGGAGATAACACTCTAAGCATAACTAAAGGT	ENF1003
	R primer	GATGTAGTTGCTTGGGACCCA	ENR1063
	Probe FAM/ZEN/IABkFQ	CCATTTTTGGTTTGGGCTTCACACCATT	ENPr1043

Table S8 Analytical validation performance specifications for the *IL1R1* RT-qPCR test [5, 15].

**Parameter**	***IL1R1* test**
Specificity (%) [1]	100
PCR Efficiency (%) (mean ± SD) [2]	96.69 ± 1.45
Linearity R^2 (mean ± SD) [2]	0.999 ± 0.001
Accuracy - RT-qPCR *vs* Next Generation Sequencing correlation (r) [3]	0.9002
Reportable Range (copies/reaction) [4]	10^2 to 10^6
Analytical sensitivity - Limit of Detection (copies/reaction) [4]	10
Analytical sensitivity - Limit of Quantification (copies/reaction) [4]	100
Precision - Inter-assay variability NCN ± SD (%CV) [5]	
HL-60	1186 ± 109 (9.2)
OCI-AML2	458 ± 59 (12.8)

[1] The specificity was verified by the absence of amplification when no reverse transcription was performed on the RNA template prior to the qPCR. The expected size and sequence of the amplicon obtained from the reaction performed on cDNA were also verified. [2] PCR efficiency and linearity were determined with independent experiments (n = 24) involving the 10-fold serial dilutions of plasmid standard (10^6 to 10^1 copies per well). Acceptable ranges are between 90 and 110% and $R^2 \geq 0.98$, respectively. [3] The accuracy of the assay was verified by comparing the results with a quantification already performed using another method: RNA sequencing (**Fig. S13**). [4] The limit of detection (LOD) is the number of copies per well that can be reliably amplified with the assay and the limit of quantification (LOQ) is the number of copies that can be reliably measured (with a low coefficient of variation). The reportable range is between the LOQ and the highest number of copies that is still measured in the linear range. [5] Precision was verified by repeating (n = 23) the measurement by RT-qPCR from a batch of RNA extracted from cell lines expressing high (HL-60) or low (OCI-AML2) levels of *IL1R1*. Acceptable performance criteria for the precision should not exceed 15% of the CV.

Table S9 Prognostic analyses of genes involved in the IL1 signaling pathway and other related genes.

**Genes**	**Overall survival**		**Relapse-free survival**	
	**HR [95% CI]**	***p* value**	**HR [95% CI]**	***p* value**
CASP1	1.00 [1.00 – 1.00]	0.53	1.00 [1.00 – 1.00]	0.61
CHUK	1.00 [0.98 – 1.02]	0.93	1.00 [0.97 – 1.02]	0.82
IKBKB	1.00 [0.9 – 1.01]	0.90	1.00 [0.99 – 1.02]	0.54
IKBKG	0.99 [0.97 – 1.01]	0.24	0.99 [0.97 – 1.02]	0.62
IL1A	1.03 [0.99 - 1.07]	0.20	1.02 [0.95 - 1.09]	0.63
IL1B	1.00 [1.00 - 1.00]	< 0.01	1.00 [1.00 - 1.001]	0.09
IL1R1	1.04 [1.03 – 1.06]	< 0.01	1.05 [1.03 – 1.07]	< 0.01
IL1R2	1.00 [1.00 – 1.00]	0.56	1.00 [1.00 – 1.00]	0.46
IL1RAP	1.013 [1.006 - 1.019]	< 0.01	1.011 [1.004 - 1.018]	< 0.01
IL1RL1	1.00 [1.00 – 1.01]	0.97	1.00 [0.99 – 1.01]	0.78
IL1RL2	1.45 [0.78 - 2.71]	0.24	2.28 [1.08 - 4.81]	0.03
IL1RN	1.00 [1.00 – 1.00]	0.60	1.00 [1.00 – 1.00]	0.76
IL18R1	1.02 [0.96 – 1.08]	0.58	0.99 [0.91 – 1.08]	0.85
IL36A	0.74 [0.09 – 6.48]	0.79	0.18 [0.01 – 3.18]	0.24
IL36B	1.02 [0.00 – Inf]	1.00	0.72 [0.00 – Inf]	0.97
IL36G	5.26 [1.29 – 21.36]	0.02	2.41 [0.10 – 55.48]	0.58
IRAK1	1.00 [0.99 – 1.00]	0.55	1.00 [0.99 – 1.01]	0.54
IRAK2	1.01 [0.99 – 1.04]	0.23	1.01 [0.97 – 1.05]	0.63
IRAK4	1.00 [0.99 – 1.02]	0.68	1.00 [0.98 – 1.02]	0.91
MAPK11	1.01 [0.99 - 1.04]	0.30	1.03 [1.00 - 1.06]	0.04
MAPK12	1.00 [0.99 - 1.02]	0.55	1.01 [0.99 - 1.02]	0.37
MAPK13	0.96 [0.89 - 1.04]	0.32	0.97 [0.88 - 1.06]	0.45
MAPK14	1.01 [0.99 - 1.01]	0.78	0.99 [0.99 - 1.01]	0.84
MLKL	1.01 [1.00 – 1.02]	0.10	1.01 [0.99 – 1.02]	0.33
MYD88	1.00 [1.00 – 1.00]	0.80	1.00 [1.00 – 1.00]	0.96
NFKB1	1.00 [1.00 – 1.01]	0.56	1.00 [1.00 – 1.01]	0.53
NFKB2	1.00 [0.99 – 1.00]	0.15	1.00 [0.99 – 1.00]	0.23
NLRC4	1.00 [0.98 – 1.03]	0.78	1.01 [0.97 – 1.04]	0.79
NLRP1	0.99 [0.99 – 1.00]	0.15	1.00 [0.99 – 1.01]	0.33
NLRP3	1.002 [0.999 - 1.004]	0.06	1.003 [1.00 - 1.005]	0.03
NLRP6	0.79 [0.64 - 0.99]	0.04	0.90 [0.75 - 1.08]	0.27
PYCARD	1.00 [1.00 – 1.00]	0.81	1.00 [1.00 – 1.00]	0.40
TNF	1.00 [1.00 – 1.01]	0.04	1.00 [1.00 – 1.01]	0.81
RIPK1	0.99 [0.95 – 1.02]	0.42	0.98 [0.94 – 1.02]	0.29
RIPK3	0.99 [0.98 – 1.01]	0.44	0.99 [0.97 – 1.01]	0.44
TNFRSF1A	1.004 [1.00 – 1.01]	0.08	1.004 [1.00 – 1.01]	0.10
TNFRSF1B	1.00 [1.00 – 1.00]	0.88	1.00 [1.00 – 1.00]	0.47
TRAF6	0.98 [0.95 – 1.01]	0.19	0.98 [0.95 – 1.02]	0.32

Analyses with censoring at time of HSCT in CR1. Gene were evaluated as continuous variables in these analyses. When gene expression is evaluated as a continuous variable, the hazard ratio (HR) depends on the range of expression for each gene. Genes with a very wide range of expression may have a statistically significant association with overall survival and/or relapse-free survival even if the HR is very close to 1.00 (e.g. *IL1B* which has a range of expression between 1.99 and 7223.32 TPM). Abbreviations: HSCT, allogeneic hematopoietic stem cell transplantation; CR1, first complete remission.

3. Supplementary Figures

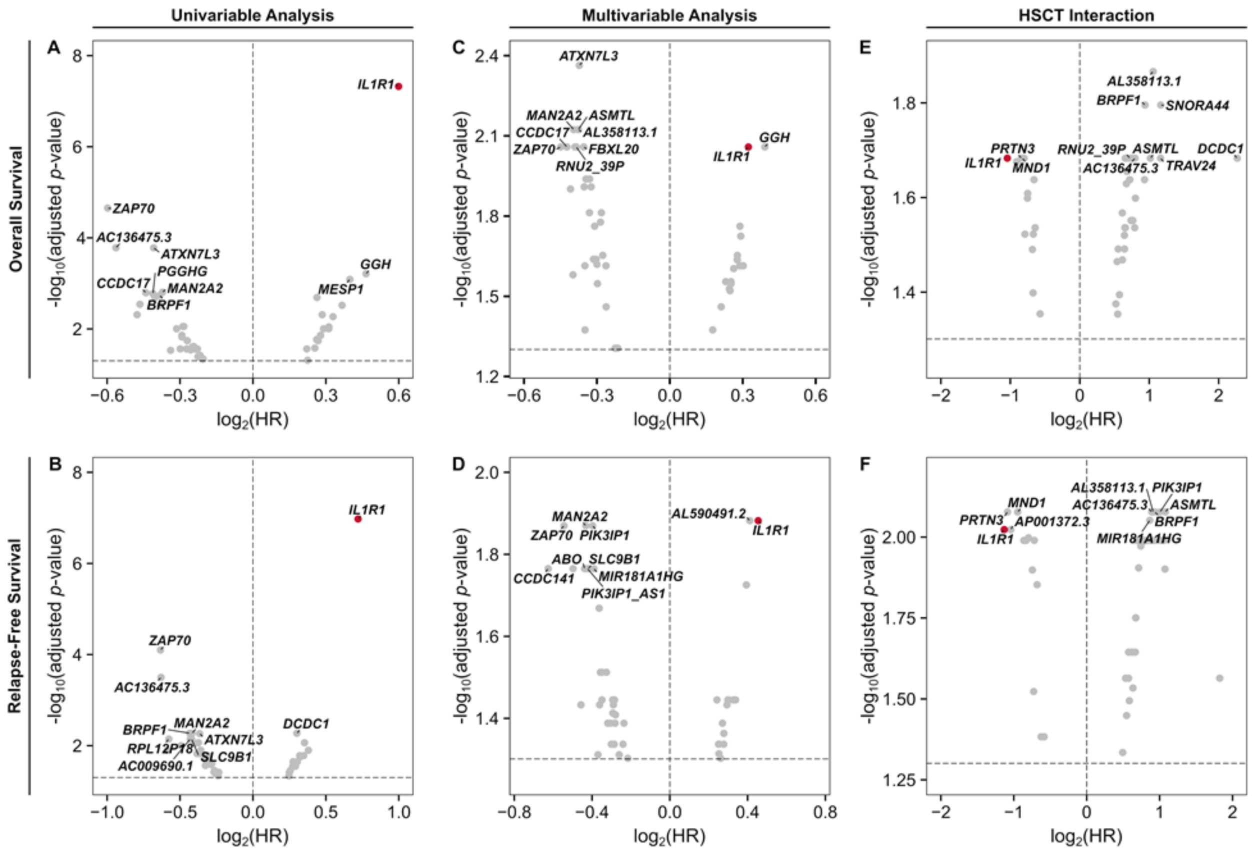

Fig. S1 Identification of *IL1R1* expression as a prognostic and predictive biomarker.

Volcano plots showing results of analyses performed to identify *IL1R1* as our leading gene expression prognostic and predictive biomarker. Results from univariable analysis (UVA) of gene expression with **A** OS **B** RFS; multivariable analysis (MVA) of gene expression with **C** OS and **D** RFS and analysis of interaction terms between gene expression and HSCT in CR1 as a time-dependent variable (HSCT-TD) for **E** OS and **F** RFS. Analyses were performed with gene expression of all protein-coding genes and long non-coding RNAs, excluding all genes with minimal expression for which all patient samples had an expression below 1.0 TPM. Hazard ratio (HR) (log_2HR) and associated adjusted *p*-value (-log_{10}(adjusted *p*-value)) are displayed on X and Y axis, respectively. Only genes that are significant for both OS and RFS in UVA and MVA and with a significant interaction with HSCT-TD ($p < 0.05$) were selected (48 genes). Among these genes, *IL1R1* (marked with red dot) is identified as the gene with the best characteristics in UVA, MVA and HSCT interaction. The names of the top 10 genes (lowest adjusted *p*-value) are displayed in each volcano plot. Horizontal line represents an adjusted *p*-value of 0.05. *p*-values were adjusted with Benjamini-Hochberg method. Age, white blood cell count and *NPM1*, *FLT3*-ITD, *DNMT3A*, bZIP *CEBPA*, *ASXL1* and *RUNX1* mutations were included as covariables in MVA.

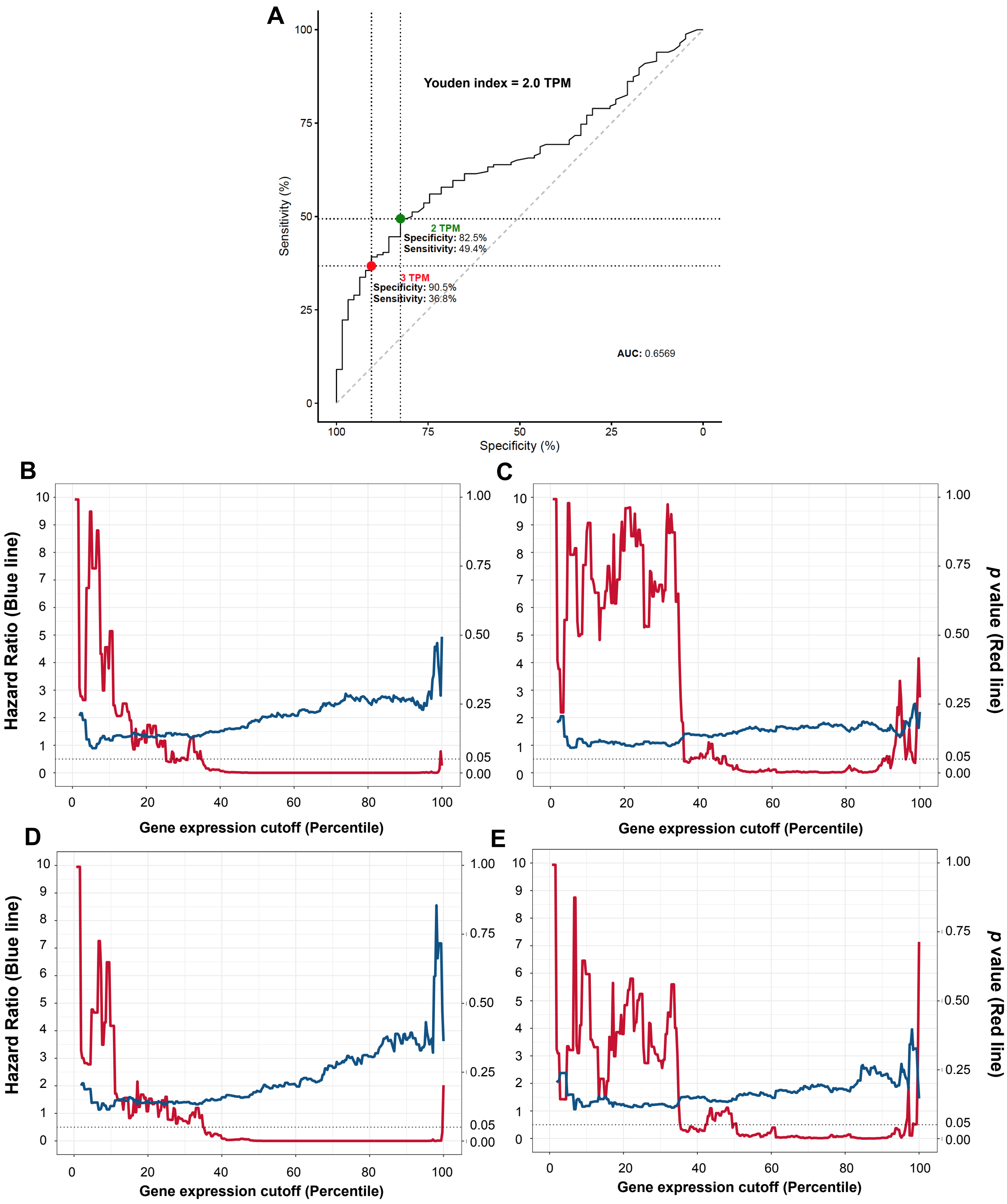

Fig. S2 Identification of the optimal cutoff value for dichotomization of *IL1R1* expression.

A Receiver operating characteristic (ROC) curve representing sensitivity (Y-axis) and specificity (X-axis) to predict 3-year overall survival censored at HSCT with all possible cutoff of *IL1R1* expression. The Youden index for the optimal cutoff of *IL1R1* was at 2.0 TPM. **B**-**E** HR and *p* values according to all possible cutoffs of *IL1R1* expression were plotted to identify the range of cutoffs for which *IL1R1* expression was significantly associated with OS and RFS in CPH models. Blue lines represent the HR values (graduation on the left Y-axis) and red

lines represent *p* values (graduation on the right Y-axis). **B** UVA for OS with all *IL1R1* cutoffs. Cutoffs between the 25th and 99th percentiles were significant with HRs ranging from 1.37 to 4.94. **C** MVA for OS with all *IL1R1* cutoffs. Cut-offs between the 36th and 98th percentiles were significant with HRs ranging from 1.38 to 2.51. **D** UVA for RFS with all *IL1R1* cutoffs. Cut-offs between the 35th and 99th percentiles were significant with HRs ranging from 1.43 to 8.55. **E** MVA for RFS with all *IL1R1* cutoffs. Cut-offs between the 35th and 98th percentiles were significant with HRs ranging from 1.45 to 3.96. For all UVA and MVA analyses, OS and RFS were censored at HSCT in first complete remission. Abbreviations: HR, hazard ratio; MVA, multivariable analysis; RFS, relapse-free survival; TPM, transcripts per million; OS, overall survival; UVA, univariable analysis; HSCT, allogeneic hematopoietic stem cell transplantation; TD, time-dependent; CPH, Cox proportional Hazard; CR1, first complete remission.

Subgroup	Nb patients (%)	HR [95% CI]	p value Univariate	p value Interaction
**Overall**	66/316 (21)	0.49 [0.33 – 0.74]	< 0.01	
**10th percentile**				
**< 0.25 TPM (low)**	6/31 (19)	0.76 [0.21 – 2.81]	0.68	7.58 x 10^{-4}
**≥ 0.25 TPM (high)**	60/285 (21)	0.48 [0.31 – 0.74]	< 0.01	
**20th percentile**				
**< 0.49 TPM (low)**	15/63 (24)	1.13 [0.52 – 2.45]	0.76	1.53 x 10^{-4}
**≥ 0.49 TPM (high)**	51/253 (20)	0.40 [0.25 – 0.64]	< 0.01	
**30th percentile**				
**< 0.77 TPM (low)**	22/95 (23)	1.05 [0.56 – 1.99]	0.88	7.46 x 10^{-5}
**≥ 0.77 TPM (high)**	44/221 (20)	0.34 [0.20 – 0.58]	< 0.01	
**40th percentile**				
**< 1.09 TPM (low)**	28/126 (22)	1.01 [0.57 – 1.78]	0.98	3.72 x 10^{-5}
**≥ 1.09 TPM (high)**	38/190 (20)	0.29 [0.16 – 0.52]	< 0.01	
**50th percentile**				
**< 1.44 TPM (low)**	34/158 (22)	0.79 [0.46 – 1.36]	0.40	8.30 x 10^{-5}
**≥ 1.44 TPM (high)**	32/158 (20)	0.29 [0.16 – 0.54]	< 0.01	
**60th percentile**				
**< 1.98 TPM (low)**	39/189 (21)	0.72 [0.43 – 1.20]	0.21	5.79 x 10^{-5}
**≥ 1.98 TPM (high)**	27/127 (21)	0.26 [0.14 – 0.50]	< 0.01	
**70th percentile**				
**< 2.37 TPM (low)**	49/220 (22)	0.69 [0.43 – 1.10]	0.12	8.61 x 10^{-5}
**≥ 2.37 TPM (high)**	17/96 (18)	0.19 [0.08 – 0.44]	< 0.01	
**80th percentile**				
**< 3.89 TPM (low)**	57/252 (23)	0.62 [0.40 – 0.97]	0.04	7.59 x 10^{-4}
**≥ 3.89 TPM (high)**	9/64 (14)	0.12 [0.04 – 0.42]	< 0.01	
**90th percentile**				
**< 6.22 TPM (low)**	63/284 (22)	0.54 [0.36 – 0.83]	< 0.01	NA
**≥ 6.22 TPM (high)**	3/32 (9)	0.09 [0.01 – 0.69]	0.02	

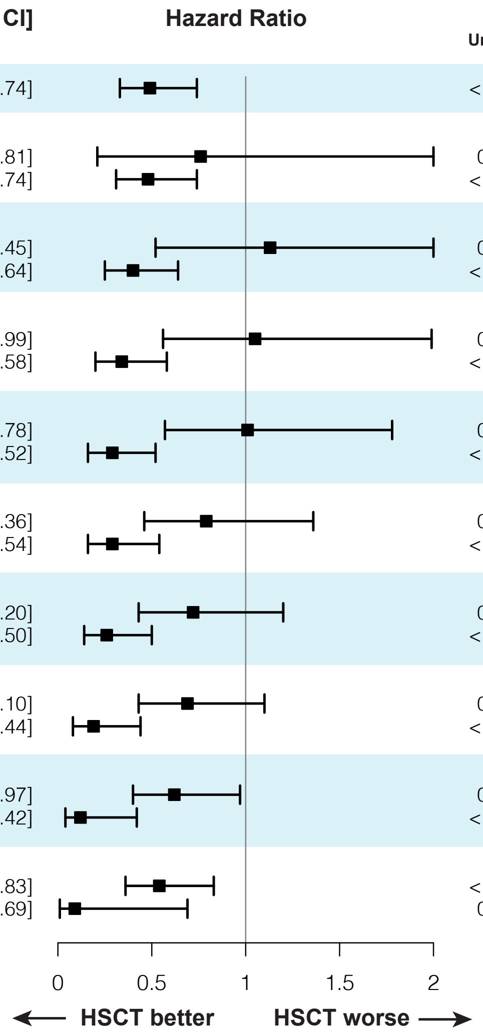

Fig. S3 Benefit from HSCT in CR1 for OS using different cutoffs for *IL1R1* expression.

The HRs for the impact of HSCT in CR1 on OS in patients with IRC AML were calculated using 9 different cutoffs for dichotomization of *IL1R1* expression. For each cutoff, the HRs are represented for subgroups of patients with low and high expression of *IL1R1*. With increasing cutoffs of *IL1R1*, the HR for HSCT in CR1 were consistently decreasing suggesting an increasing benefit from HSCT in CR1 with increasing levels of *IL1R1* expression. The number and proportion of patients who have undergone HSCT in CR1 is represented for each group in the second column. Similar results were obtained when using RFS as the clinical endpoint (data not shown). Abbreviations: HSCT, allogeneic hematopoietic stem cell transplantation; CR1, first complete remission HR, hazard ratio; OS, overall survival; IRC, intermediate-risk cytogenetics; RFS, relapse-free survival; TPM, transcripts per million.

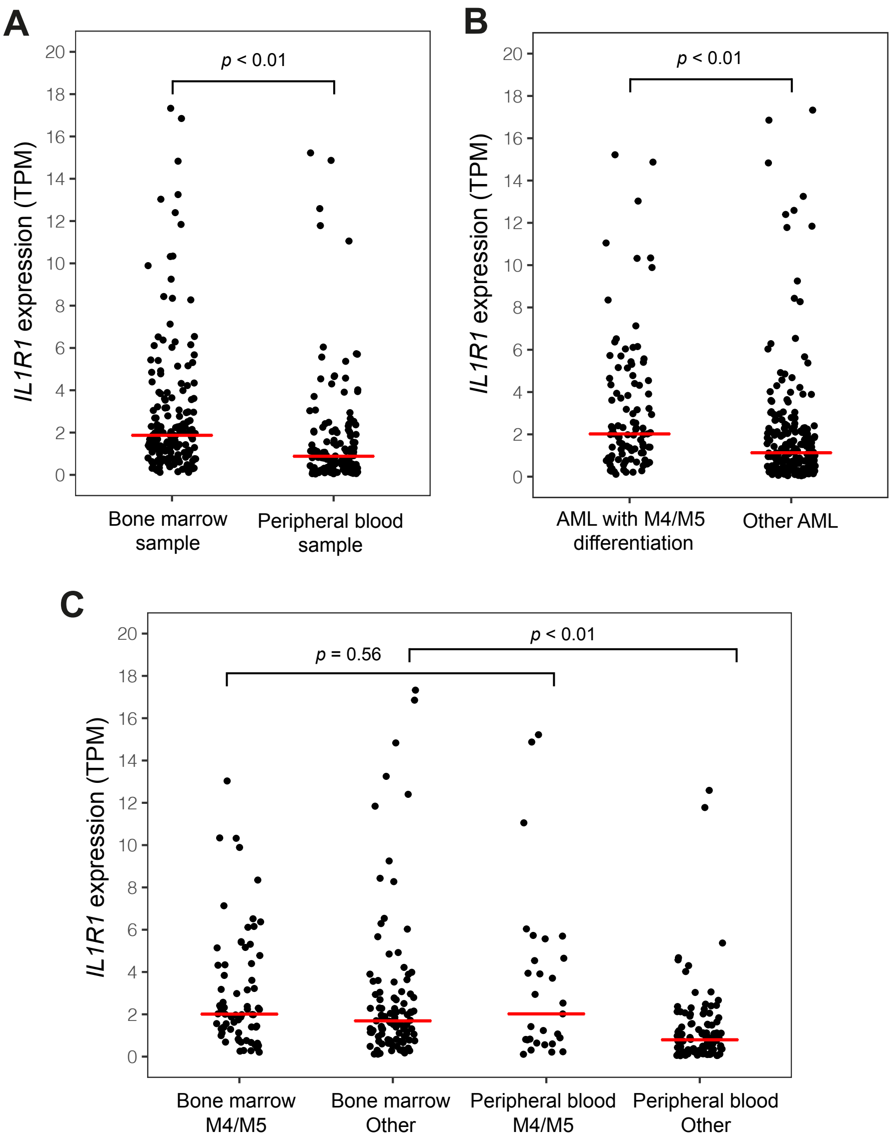

Fig. S4 Expression of *IL1R1* according to the type of sample sequenced and myelomonocytic differentiation of the AML

In the Leucegene IRC AML cohort, 137 (43%) patients had a peripheral blood (PB) sample sequenced and 179 (57%) had a bone marrow (BM) sequenced for whole transcriptomics (RNA-sequencing). Expression of *IL1R1* is plotted according to the type of sample sequenced and AML with myelomonocytic differentiation (French-American-British [FAB] classification M4 or M5). In each panel, *IL1R1* expression is presented in TPM, each black dot represents a patient sample, and the horizontal red lines represent the median of *IL1R1* expression in each subgroup. **A** *IL1R1* expression according to the type of sample sequenced. In patients who had a bone marrow sample sequenced, the median *IL1R1* expression was 1.96 TPM (IQR, 1.07 – 3.74) versus 0.89 TPM (IQR, 0.31 – 2.07) in patients who had a peripheral blood sample sequenced ($p < 0.01$). **B** *IL1R1* expression according to the presence of M4/M5 AML. In patients with M4/M5 AML, the median *IL1R1* expression was 2.02 TPM (IQR, 0.99– 4.65) versus 1.17 TPM (IQR, 0.49 – 2.29) in patients without M4/M5 AML ($p < 0.01$). **C** The difference in *IL1R1* expression between BM and PB samples was only observed in patients without M4/M5 AML (median BM 1.72 TPM vs BP 0.82 TPM, p < 0.01), but not in patients with M4/M5 AML (median BM 2.05 TPM vs 2.02 TPM, $p = 0.56$). Abbreviations: TPM, transcripts per million; IQR, interquartile range.

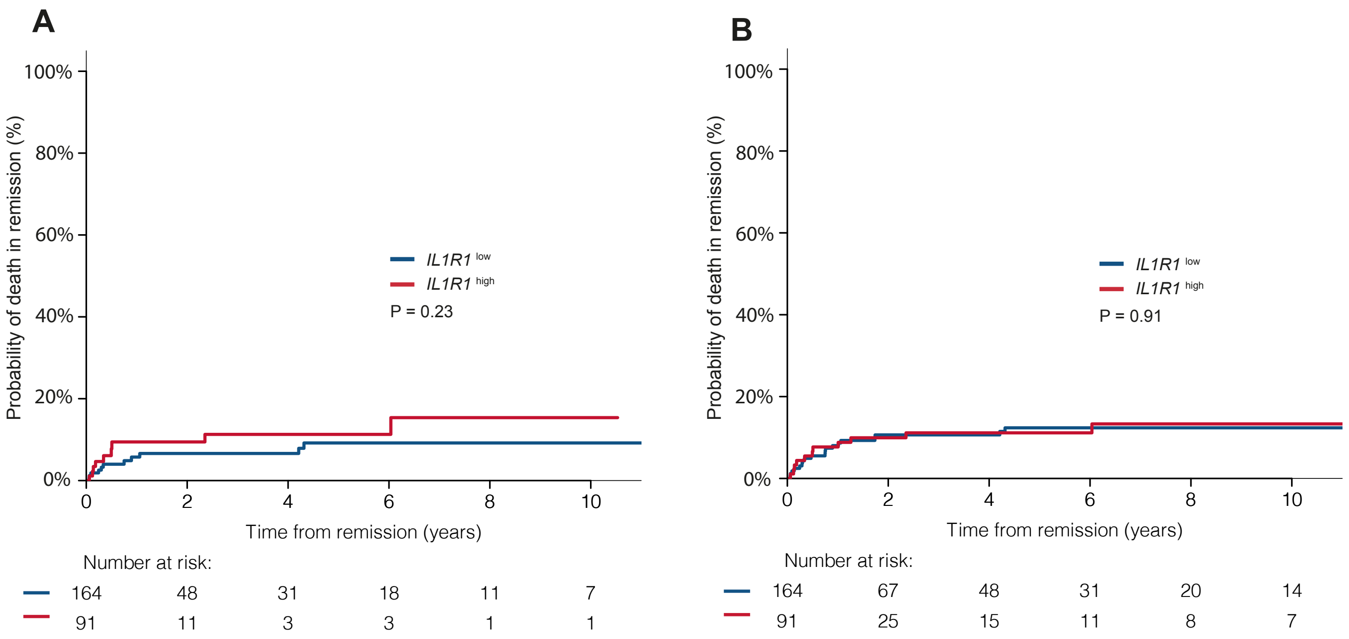

Fig. S5 Cumulative incidence of death in remission according to *IL1R1* expression.

Cumulative incidence of death in remission (CID) **A** with censoring at time of HSCT in CR1 and **B** without censoring at time of HSCT in CR1. **A** At 5 years, CID censored at HSCT was 11% in patients with *IL1R1*^{low} and 9% in patients with *IL1R1*^{high}. **B** At 5 years, CID (without censoring at HSCT) was 12% in patients with *IL1R1*^{low} and 11% in patients with *IL1R1*^{high}. Abbreviations: CID, cumulative incidence of death in remission; HSCT, allogeneic hematopoietic stem cell transplantation; CR1, first complete remission. *IL1R1*^{low} (blue line); *IL1R1*^{high} (red line).

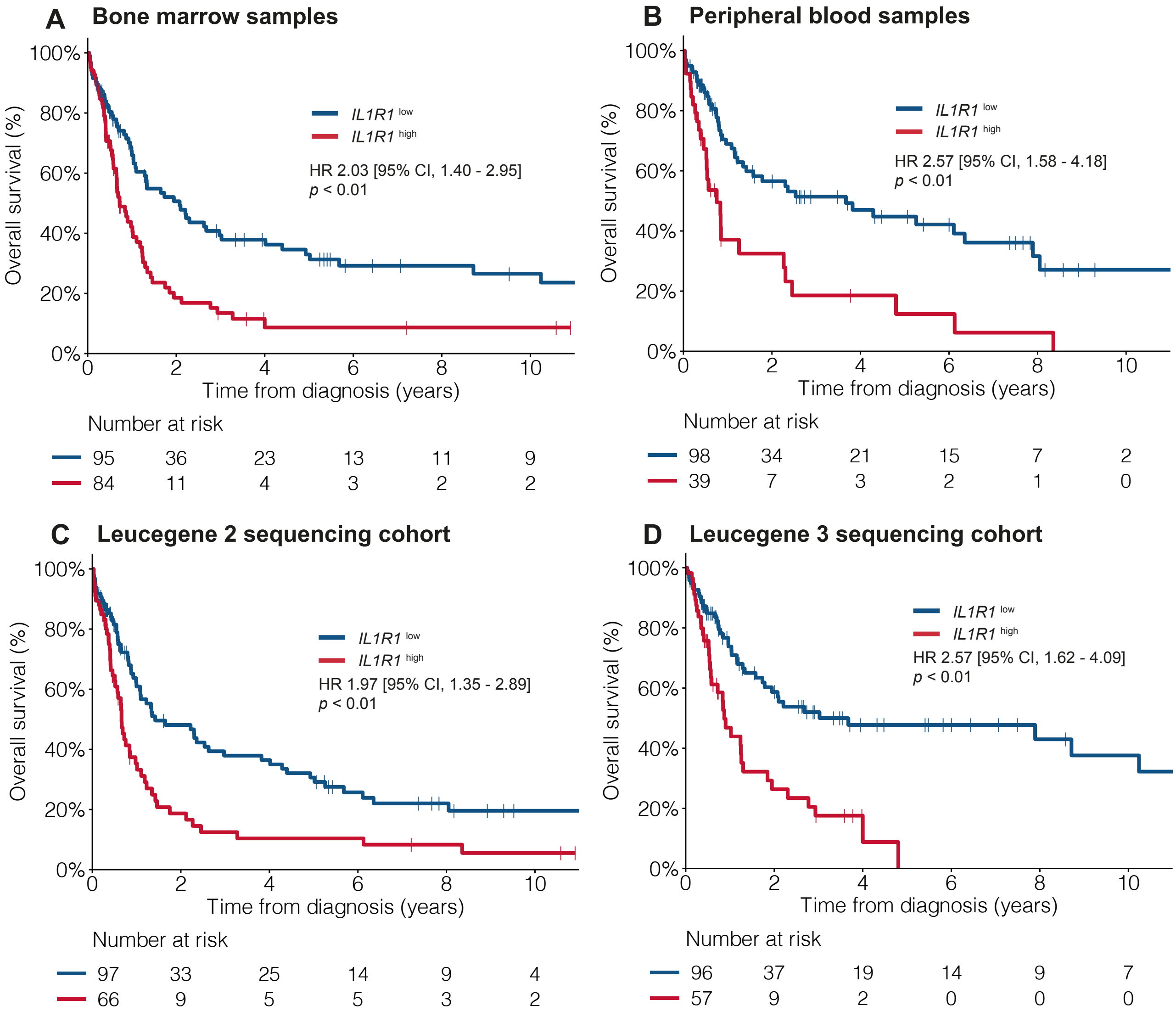

Fig. S6 Prognostic impact of *IL1R1* expression according to the type of sample sequenced and the sequencing cohort

Overall survival (OS) censored at HSCT in CR1 according to *IL1R1* expression in subgroups of patients defined by the type of sample sequenced and by the sequencing cohort **A** Impact of *IL1R1* expression on OS in patients who had a bone marrow sample sequenced. **B** Impact of *IL1R1* expression on OS in patients who had a peripheral blood sample sequenced. **C** Impact of *IL1R1* expression on OS in patients from the Leucegene 2 sequencing cohort. **D** Impact of *IL1R1* expression on OS in patients from the Leucegene 3 sequencing cohort. Abbreviations: OS, overall survival; HSCT, allogeneic hematopoietic stem cell transplantation; CR1, first complete remission. *IL1R1*^{low} (blue line); *IL1R1*^{high} (red line).

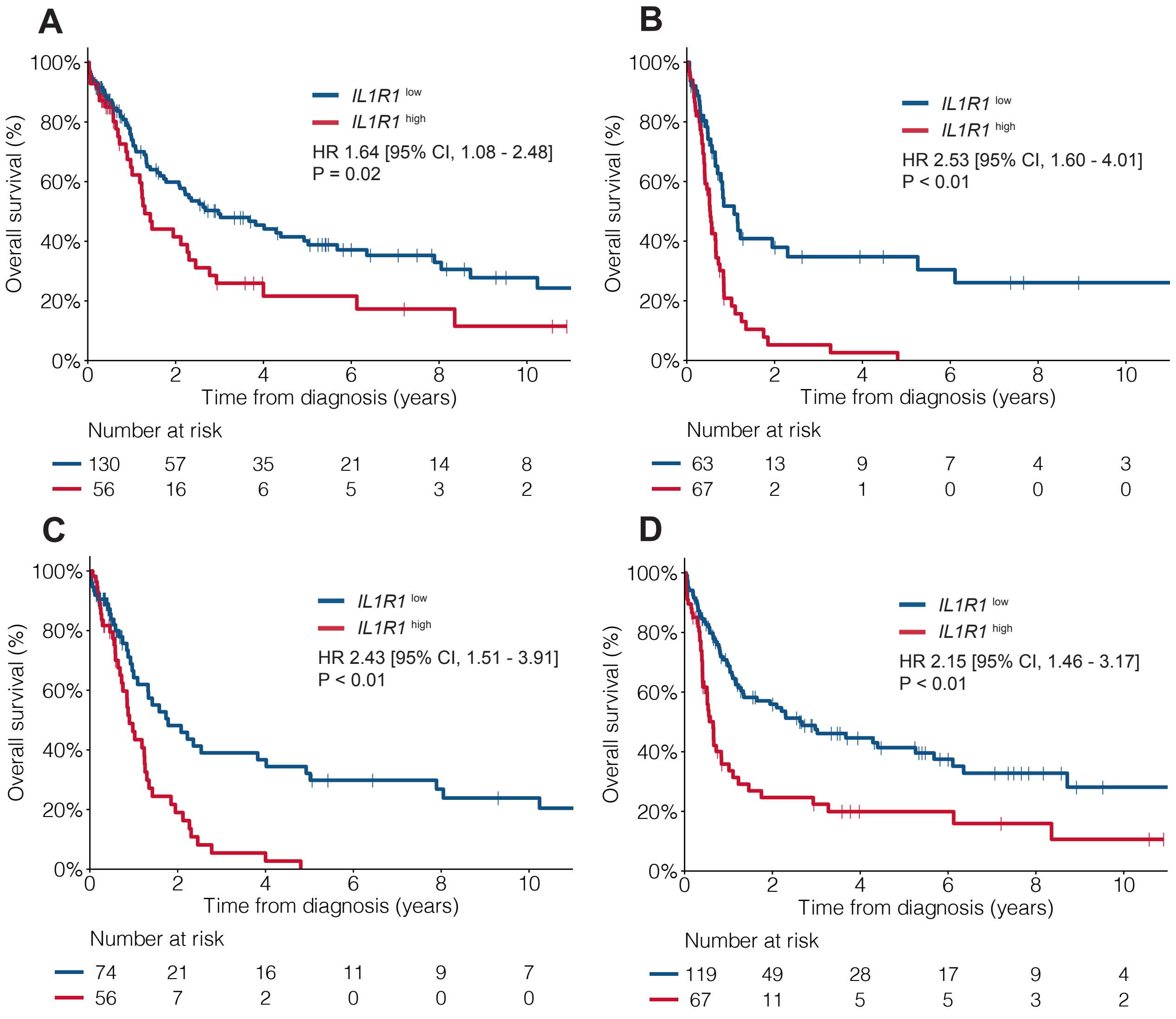

Fig. S7 Prognostic impact of *IL1R1* expression according to *NPM1* or *FLT3*-ITD mutational status.

Overall survival (OS) censored at HSCT in CR1 according to *IL1R1* expression in subgroups of patients **A** without *FLT3*-ITD mutation or **B** with *FLT3*-ITD, **C** *NPM1* wild-type, **D** *NPM1* mutation. **A** In patients without *FLT3*-ITD mutation, the 3-year OS rate was 49% and 26% in patients with *IL1R1*^{low} and *IL1R1*^{high}, respectively. **B** In patients with *FLT3*-ITD mutation, the 3-year OS rate was 35% and 5% in patients with *IL1R1*^{low} and *IL1R1*^{high}, respectively. **C** In patients without *NPM1* mutation, the 3-year OS was 39% with *IL1R1*^{low} and 5% with *IL1R1*^{high}. **D** In patients with *NPM1* mutation, the 3-year OS was 48% with *IL1R1*^{low} and 22% with *IL1R1*^{high}. Abbreviations: OS, overall survival; HSCT, allogeneic hematopoietic stem cell transplantation; CR1, first complete remission. *IL1R1*^{low} (blue line); *IL1R1*^{high} (red line).

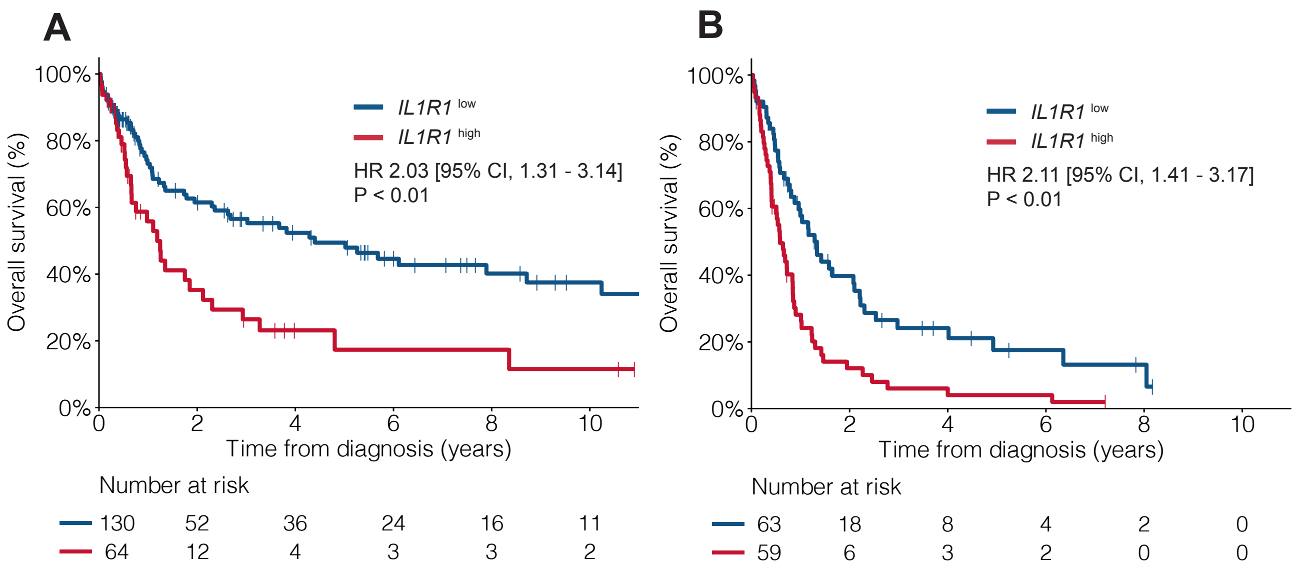

Fig. S8 Prognostic impact of *IL1R1* expression according to age.

Overall survival (OS) censored at HSCT in CR1 according to *IL1R1* expression in subgroups of **A** patients aged < 60 years and **B** patients aged ≥ 60 years. **A** In patients < 60 years old, the 3-year OS was 57% with *IL1R1*^{low} and 26% with *IL1R1*^{high} respectively. **B** In patients ≥ 60 years old, the 3-year OS was 24% with *IL1R1*^{low} and 6% with *IL1R1*^{high} respectively. Abbreviations: OS, overall survival; HSCT, allogeneic hematopoietic stem cell transplantation; CR1, first complete remission. *IL1R1*^{low} (blue line); *IL1R1*^{high} (red line).

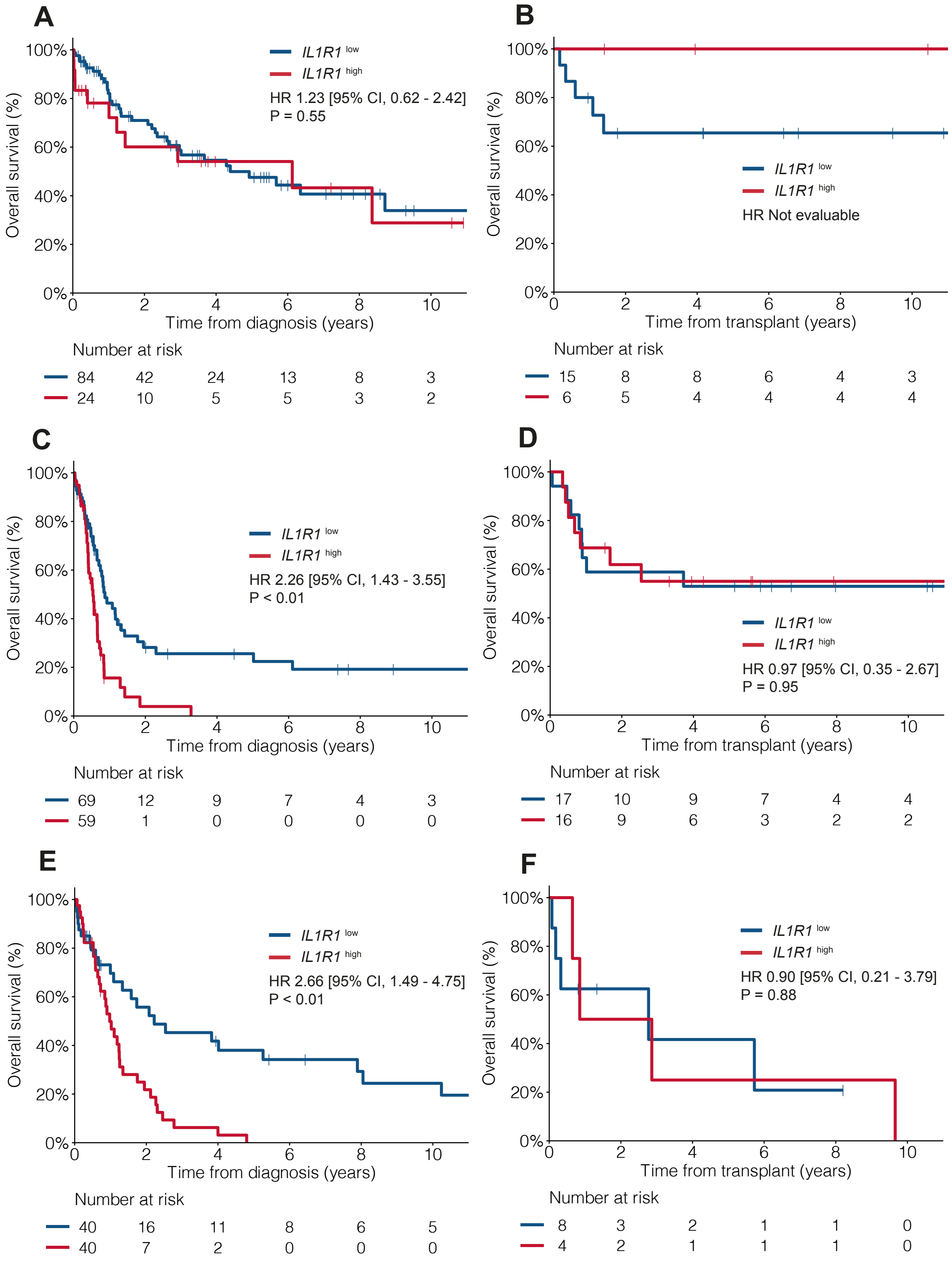

Fig. S9 Prognostic impact of *IL1R1* expression according to 2022 ELN risk classification.

Overall survival (OS) censored at HSCT in CR1 according to *IL1R1* expression in subgroups of patients with ELN 2022 **A** favorable risk, **C** intermediate risk, and **E** adverse risk [14]. OS post-HSCT in CR1 according to *IL1R1* expression in subgroups of patients with ELN 2022 **B** favorable risk, **D** intermediate risk, and **F** adverse risk. Abbreviations: OS, overall survival; HSCT, allogeneic hematopoietic stem cell transplantation; CR1, first complete remission; ELN, European LeukemiaNet. *IL1R1*^{low} (blue line); *IL1R1*^{high} (red line).

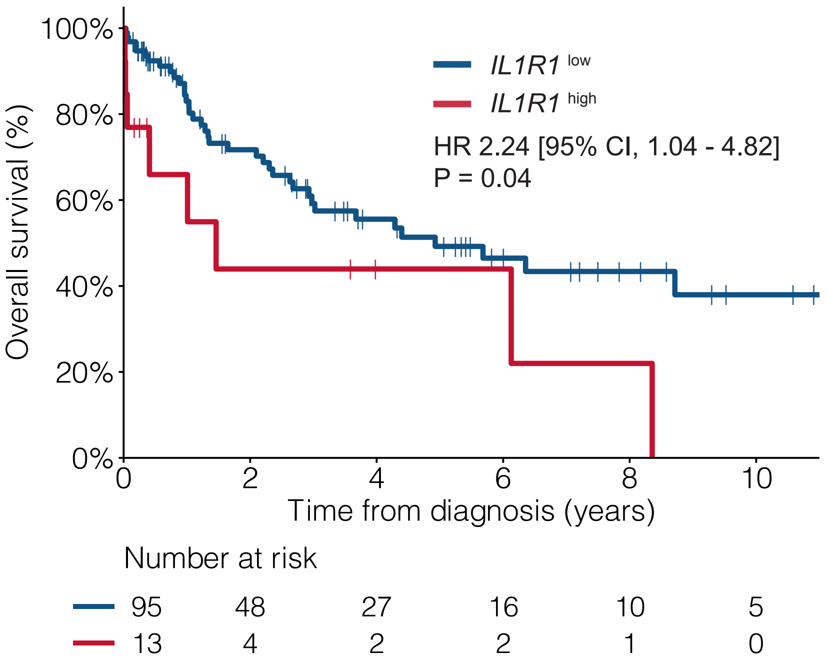

Fig S10 Prognostic impact of *IL1R1* with a higher cutoff value in patients with ELN 2022 favorable-risk AML.

Overall survival according to *IL1R1* expression in patients with favorable risk AML according to ELN 2022 with a higher cutoff for *IL1R1* expression [14]. The cutoff used for these analyses was the 3rd quartile (or 75th percentile) which corresponds to 3.0 TPM. Abbreviations: ELN, European LeukemiaNet; TPM, transcripts per million. *IL1R1*^{low} (blue line); *IL1R1*^{high} (red line).

Subgroup	Nb patients (%)	HR [95% CI]	p value Univariate	p value Interaction
**Overall**	66/255 (26)	0.57 [0.39 – 0.84]	< 0.01	
**Age**				
**< 60 y.o.**	59/163 (36)	0.75 [0.48 – 1.16]	0.20	0.31
**≥ 60 y.o.**	7/92 (8)	0.4 [0.12 – 1.29]	0.13	
**Sex**				
**Male**	33/135 (28)	0.58 [0.33 – 1.01]	0.05	0.90
**Female**	33/120 (24)	0.6 [0.35 – 1.06]	0.08	
**WBC**				
**< 50 x 10^9/L**	38/153 (25)	0.66 [0.4 – 1.02]	0.11	0.12
**≥ 50 x 10^9/L**	28/101 (28)	0.45 [0.24 – 0.85]	0.01	
NPM1				
**Mutated**	28/154 (18)	0.53 [0.28 – 0.99]	0.05	0.94
**Wild-type**	38/101 (38)	0.49 [0.29 – 0.82]	< 0.01	
***FLT3*-ITD**				
**Mutated**	30/100 (30)	0.34 [0.18 – 0.65]	< 0.01	< 0.01
**Negative**	36/155 (23)	0.77 [0.47 – 1.26]	0.30	
**bZIP *CEBPA***				
**Mutated**	10/16 (63)	0.75 [0.1 – 5.82]	0.79	0.84
**Negative**	56/239 (23)	0.63 [0.42 – 0.94]	0.02	
**MRGM**				
**Presence**	14/69 (21)	0.53 [0.25 – 1.1]	0.09	0.92
**Absence**	52/186 (28)	0.62 [0.39 – 0.99]	0.04	
DNMT3A				
**Mutated**	14/102 (14)	0.61 [0.29 – 1.28]	0.19	0.78
**Wild-type**	52/153 (34)	0.6 [0.37 – 0.96]	0.03	
***IL1R1* expression**				
***IL1R1* low**	40/164 (24)	0.75 [0.45 – 1.23]	0.25	0.02
***IL1R1* high**	26/91 (29)	0.32 [0.17 – 0.61]	< 0.01	

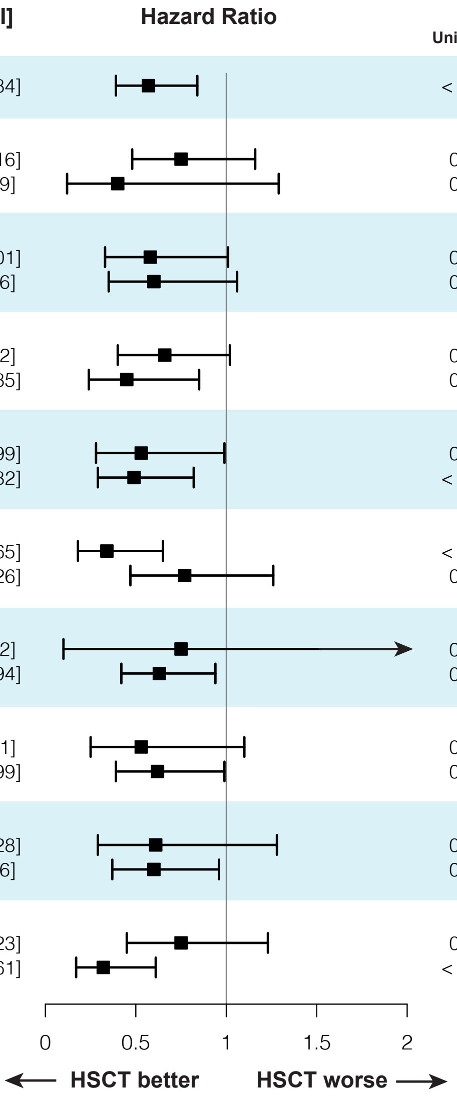

Fig. S11 Benefit from HSCT in CR1 for RFS in clinicopathological subgroups of patients.

The endpoint used for these analyses was RFS. HR and *p*-values were obtained using HSCT in CR1 as a time-dependent variable in CPH models. Interaction terms were tested between HSCT-TD and covariables in CPH models. The number and proportion of patients who have undergone HSCT in CR1 is represented for each group in the second column. Abbreviations: HSCT, allogeneic hematopoietic stem cell transplantation; CR1, first complete remission; RFS, relapse-free survival; IRC, intermediate-risk cytogenetics; HR, hazard ratio; CPH, Cox proportional Hazard; TD, time-dependent; y.o., years old; WBC, white blood cell; MRGM, myelodysplasia-related gene mutations.

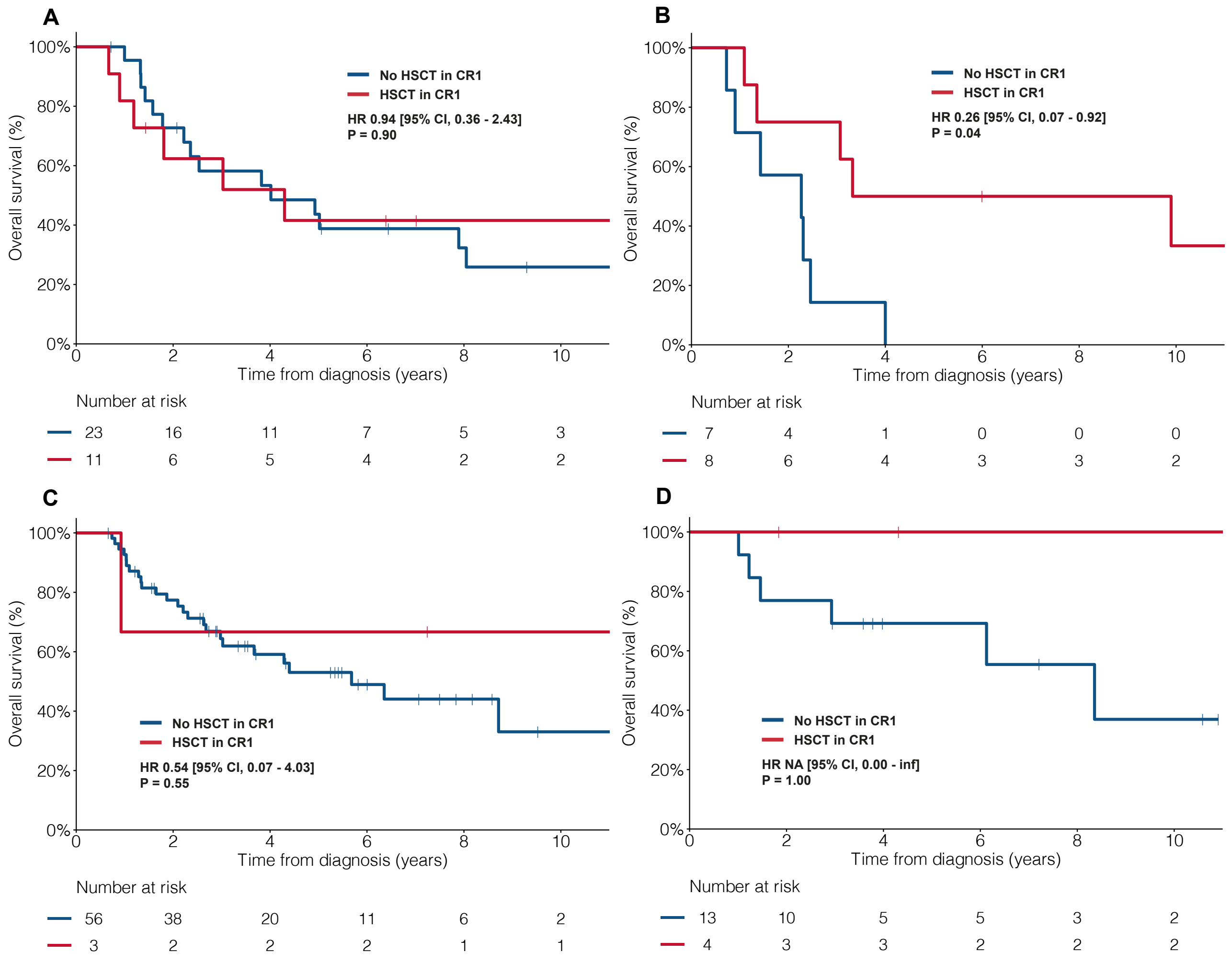

Fig. S12 Impact of HSCT in CR1 on survival outcomes according to *NPM1* mutational status and *IL1R1* expression in patients *FLT3*-ITD negative

Landmark analysis comparing survival outcomes in patients who proceeded to HSCT in CR1 (red line) versus those who did not (blue line). Landmark time was established at 6 months. **A** OS according to HSCT in CR1 in patients with *NPM1* WT / *FLT3*-ITD negative / *IL1R1*^{low}. **B** OS according to HSCT in CR1 in patients with *NPM1* WT / *FLT3*-ITD negative / *IL1R1*^{high}. **C** OS according to HSCT in CR1 in patients with *NPM1* mut / *FLT3*-ITD negative / *IL1R1*^{low}. **D** OS according to HSCT in CR1 in patients with *NPM1* mut / *FLT3*-ITD negative / *IL1R1*^{high}. Abbreviations: HSCT, allogeneic hematopoietic stem cell transplantation; CR1, first complete remission; HR, hazard ratio.

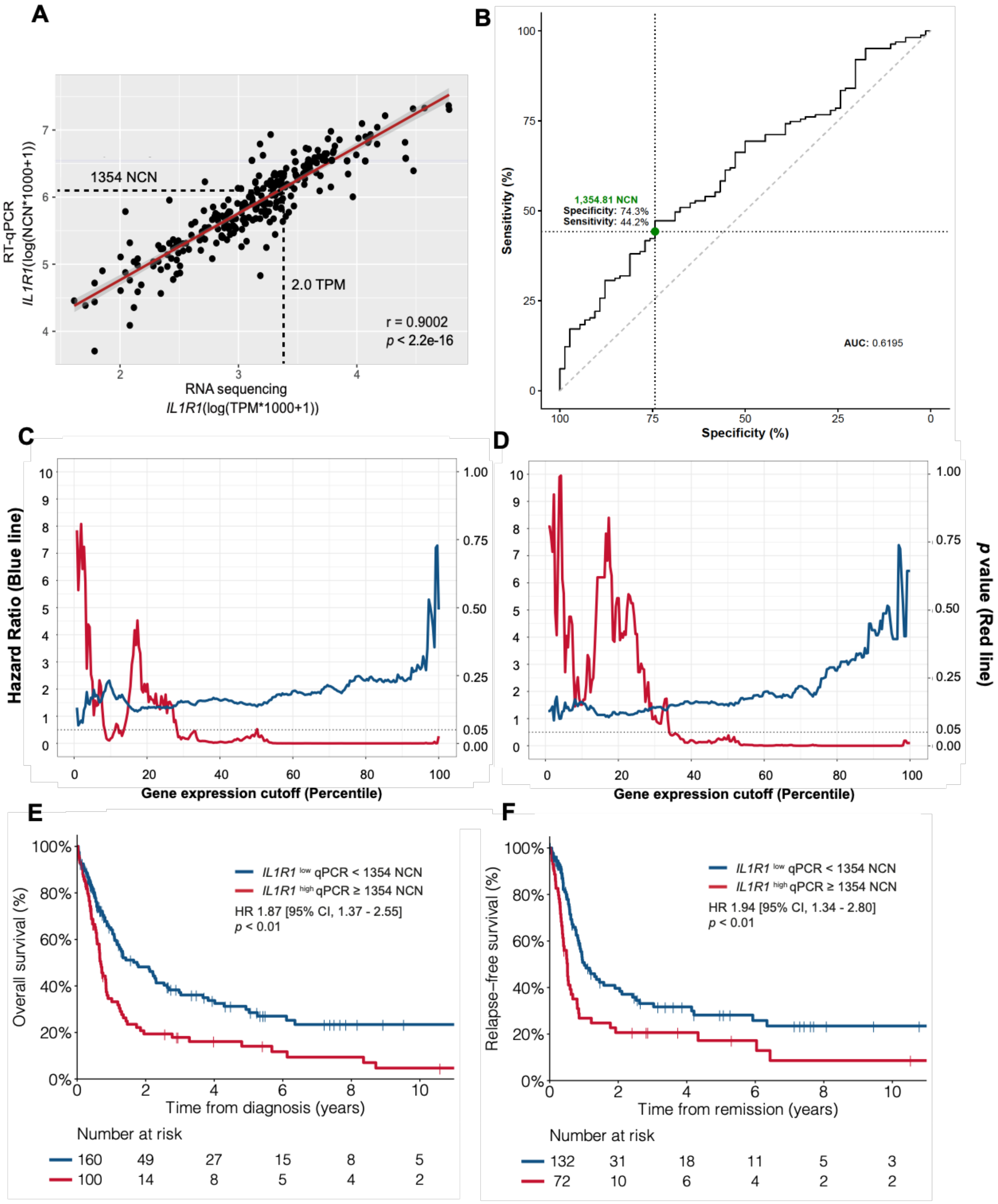

Fig. S13 Correlation between *IL1R1* expression quantification by the RT-qPCR test and RNA sequencing and clinical validation of the *IL1R1* RT-qPCR test in the Leucegene cohort.

A *IL1R1* expression was quantified with the RT-qPCR test in 260 patient samples with intermediate-risk cytogenetics AML in the Leucegene prognostic cohort. Each black dot represents a patient sample with results for *IL1R1* expression value by RT-qPCR (Y-axis) and RNA sequencing (X-axis). The comparison with the RNA sequencing data for the same patient samples shows a strong correlation between the two methods (r = 0.9002, p < 2.2e-16). Trendline in red with 95% confidence interval in gray. The *IL1R1* expression value of 2.0 TPM selected as the cutoff with RNA-sequencing corresponded approximately to a value of 1354 normalized copy number (NCN) with the RT-qPCR test for *IL1R1* expression (between 1141 and 1502 NCN depending on the use of non-transformed values or log-transformed values). **B** Receiver operating characteristic (ROC) curve representing sensitivity (Y-axis) and specificity (X-axis) to predict 3-year overall survival (OS) with all possible cutoff of *IL1R1* expression by RT-qPCR. **C**-**D** HR and *p* values according to all possible cutoffs of *IL1R1* expression by RT-qPCR to identify the range of cutoffs for which *IL1R1* expression by RT-qPCR was significantly associated with OS and RFS. Blue lines represent the HR values (graduation on the left Y-axis) and red lines represent *p* values (graduation on the right Y-axis). **C** Univariable analysis (UVA) for OS with all *IL1R1* NCN cutoffs. Cutoffs between the 28th and 100th percentiles were significant with HRs ranging from 1.39 to 7.28. **D** UVA for relapse-free survival (RFS) with all *IL1R1* NCN cutoffs. Cut-offs between the 34th and 100th percentiles were significant with HRs ranging from 1.47 to 7.39. For all analyses, OS and RFS were censored at HSCT in first complete remission. **E** OS and **F** RFS for patients with $IL1R1^{low}$ < 1354 NCN (blue line) and $IL1R1^{high}$ ≥ 1354 NCN (red line) measured by RT-qPCR. Survival times are censored at time of HSCT in CR1. Abbreviations: HR, hazard ratio; MVA, multivariable analysis; RFS, relapse-free survival; TPM, transcripts per million; OS, overall survival; UVA, univariable analysis; HSCT, allogeneic hematopoietic stem cell transplantation; TD, time-dependent; CPH, Cox proportional Hazard; CR1, first complete remission.

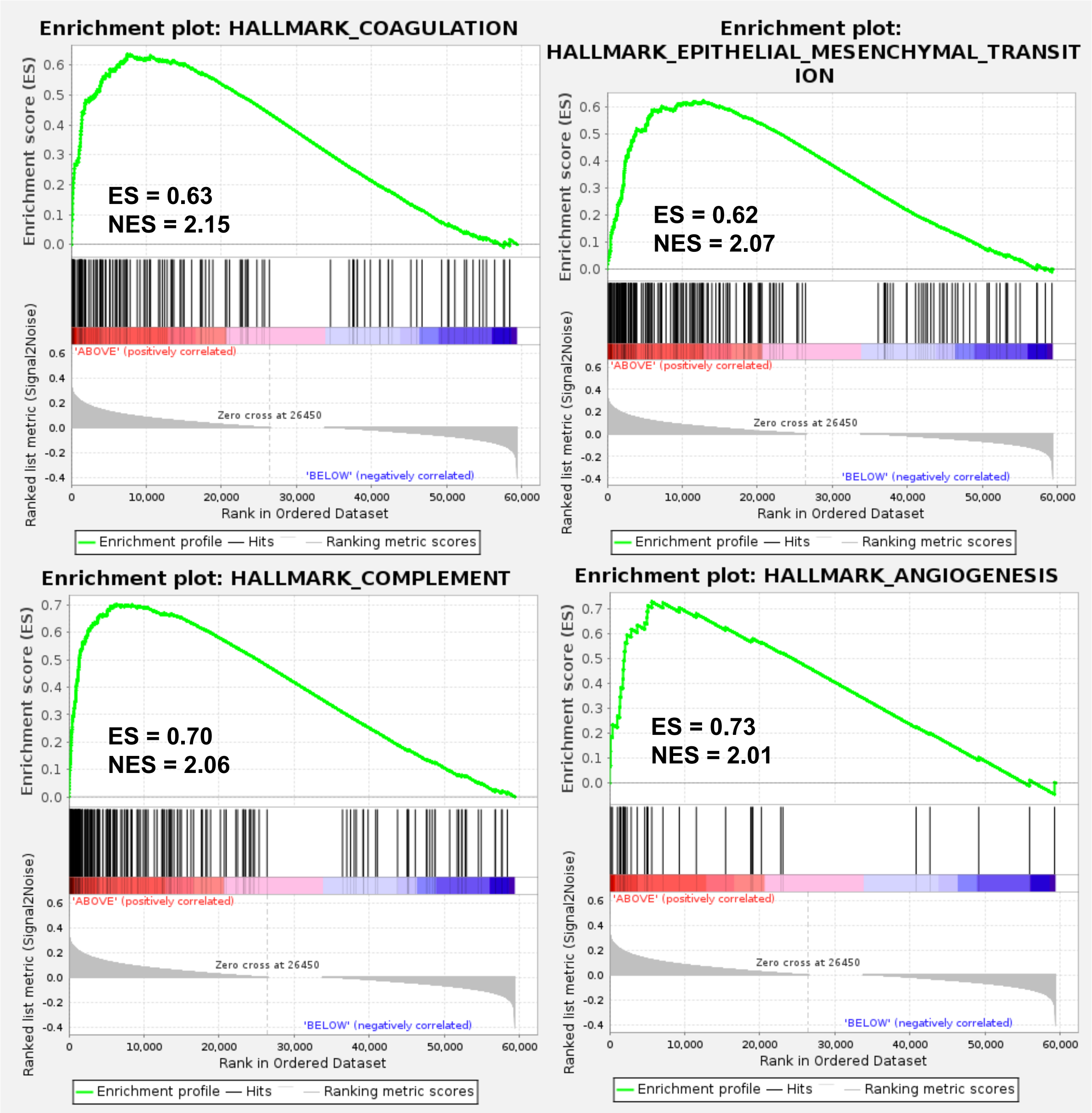

Fig. S14 Additional Gene Set Enrichment Analyses between patients with high and low expression of *IL1R1*.

Results of the GSEA comparing patients with low versus high expression of *IL1R1*. The cutoff of *IL1R1* expression for these analyses was 2.0 TPM (61st percentile), the same cutoff used for prognostic analyses and DGE analyses. These 4 enrichment plots represent 4 of the 5 top hallmark gene sets associated with high expression of *IL1R1* in the Leucegene intermediate-risk cytogenetics AML cohort. Abbreviations: GSEA, gene set enrichment analyses; TPM, transcripts per million; DGE, differential gene expression ES, enrichment score; NES, normalized enrichment score.

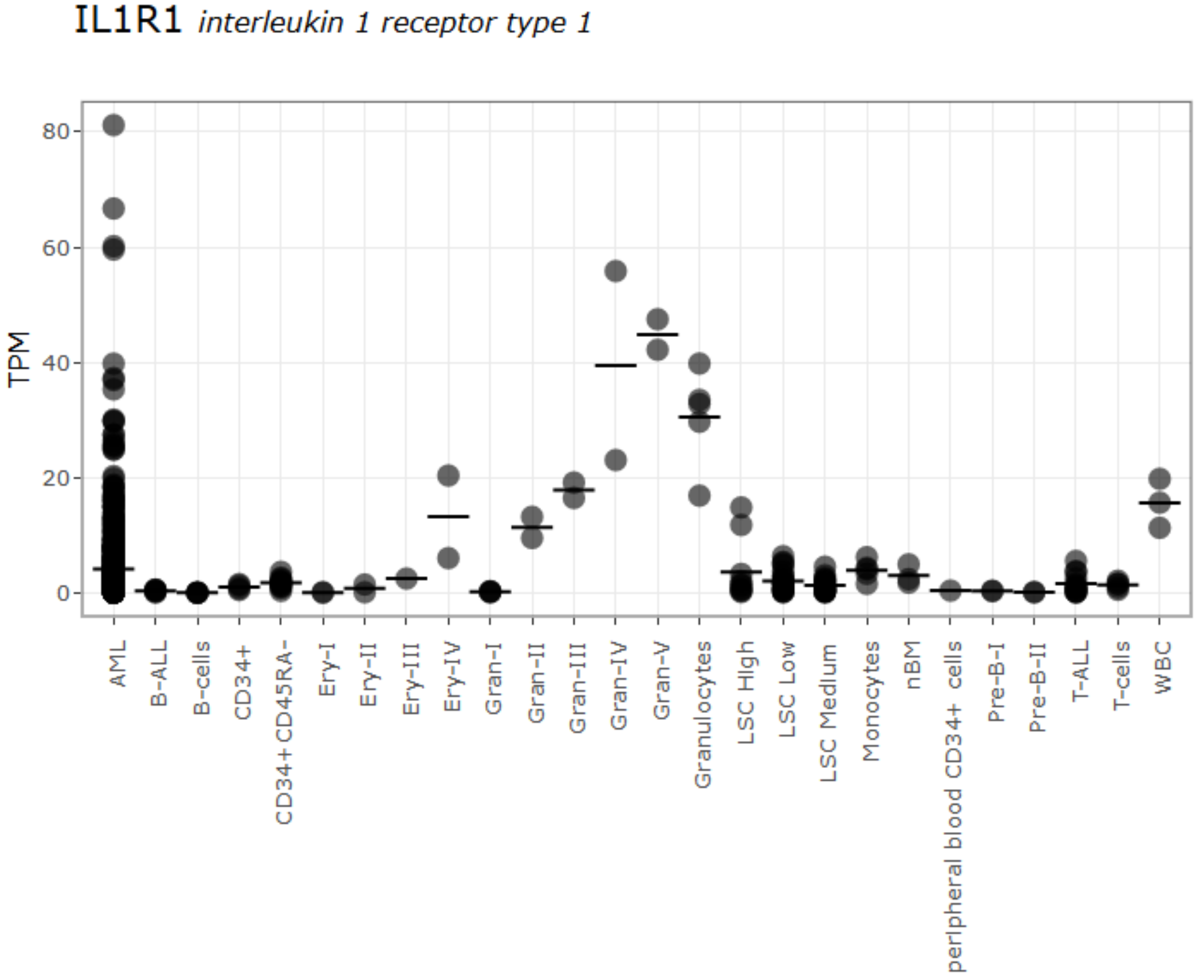

Fig. S15 Analysis of *IL1R1* expression in normal blood and bone marrow populations and in acute leukemias sequenced in the Leucegene project.

Result of *IL1R1* expression values (in TPM) obtained by bulk RNA sequencing of normal bone marrow and peripheral blood cell populations and in a subset of acute leukemias sequenced in the Leucegene project. Normal bone marrow and peripheral blood cell populations were sorted and sequenced (RNA sequencing) as previously described [16]. Abbreviations: TPM, transcripts per million; AML, acute myeloid leukemias; ALL, acute lymphoblastic leukemias; LSC, leukemia stem cell (frequency).

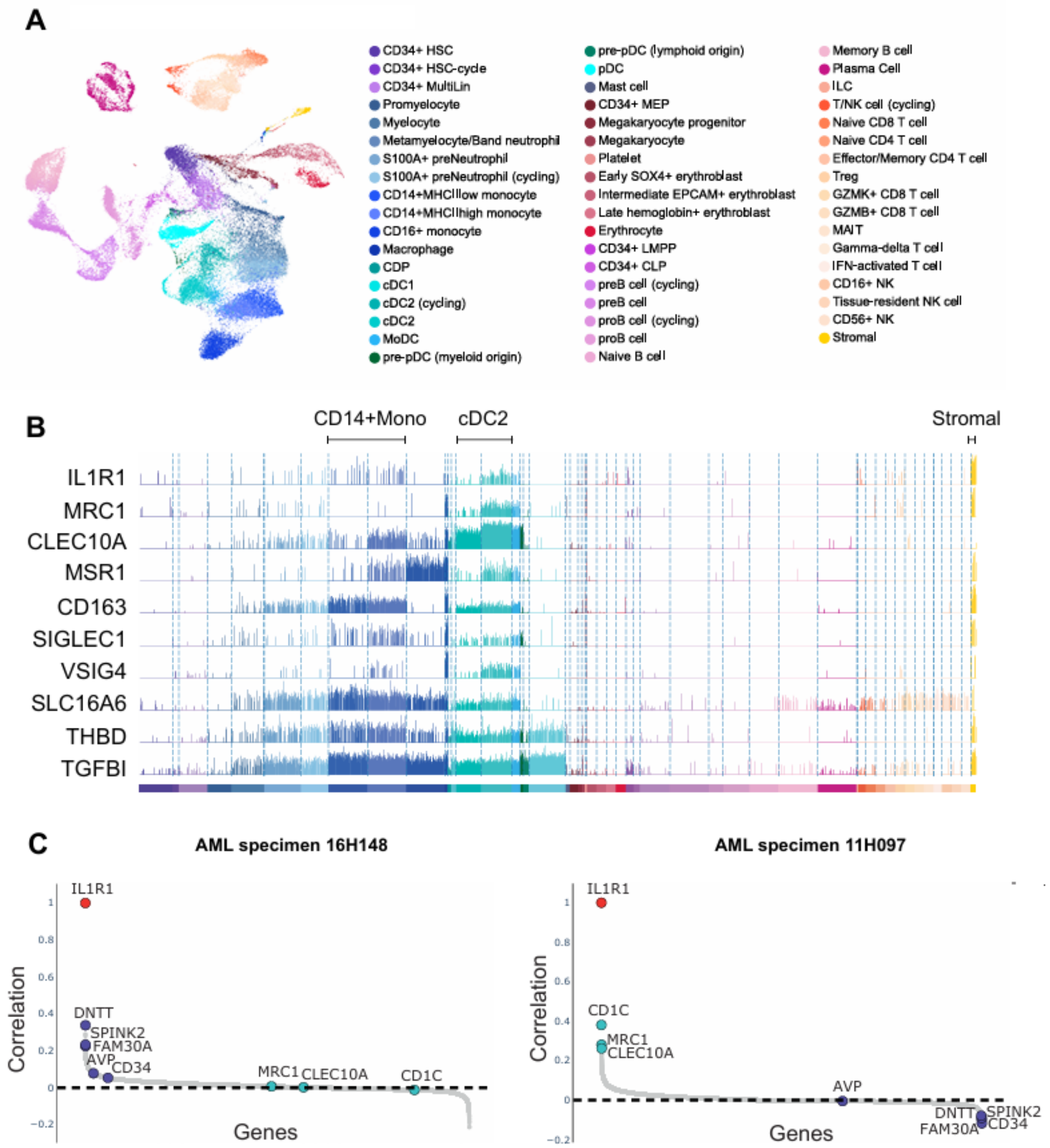

Fig. S16 Single-cell RNA sequencing of normal bone marrow and AML specimens.

A UMAP representation after Harmony integration of normal bone marrow single-cell RNA sequencing data with each cell color coded according to their cell type. **B** Marker genes expression similarly colored, with cells ordered (x axis) per cell type. CD14+ Monocytes, cDC2 and stromal cells (highlighted) express *IL1R1*. **C** Waterfall plot showing that in sample 16H148, *IL1R1* expression is highly correlated with genes associated with an immature phenotype such as *DNTT, SPINK2, AVP, CD34* and *FAM30A* and anticorrelated with the expression of genes associated with cDC2 such as *MRC1, CLEC10A and CD1C*. **D** In sample 11H097, *IL1R1* expression is correlated with that of genes associated with cDC2 and anticorrelated with that of genes associated with immature blasts. Abbreviation: cDC2, conventional dendritic cells 2.

References

1. Bray NL, Pimentel H, Melsted P, Pachter L. Near-optimal probabilistic RNA-seq quantification. Nat Biotechnol. 2016;34(5):525-7.
2. Lavallee VP, Baccelli I, Krosl J, Wilhelm B, Barabe F, Gendron P, et al. The transcriptomic landscape and directed chemical interrogation of MLL-rearranged acute myeloid leukemias. Nat Genet. 2015;47(9):1030-7.
3. Audemard EO, Gendron P, Feghaly A, Lavallee VP, Hebert J, Sauvageau G, et al. Targeted variant detection using unaligned RNA-Seq reads. Life Sci Alliance. 2019;2(4).
4. Zwiener I, Frisch B, Binder H. Transforming RNA-Seq data to improve the performance of prognostic gene signatures. PLoS One. 2014;9(1):e85150.
5. Bustin SA, Benes V, Garson JA, Hellemans J, Huggett J, Kubista M, et al. The MIQE guidelines: minimum information for publication of quantitative real-time PCR experiments. Clin Chem. 2009;55(4):611-22.
6. Beillard E, Pallisgaard N, van der Velden VH, Bi W, Dee R, van der Schoot E, et al. Evaluation of candidate control genes for diagnosis and residual disease detection in leukemic patients using 'real-time' quantitative reverse-transcriptase polymerase chain reaction (RQ-PCR) - a Europe against cancer program. Leukemia. 2003;17(12):2474-86.
7. Marquis M, Beaubois C, Lavallee VP, Abrahamowicz M, Danieli C, Lemieux S, et al. High expression of HMGA2 independently predicts poor clinical outcomes in acute myeloid leukemia. Blood Cancer J. 2018;8(8):68.
8. Budd JR, Durham AP, Gwise TE, Iriarte B, Kallner A, Linnet K, et al. CLSI document EP09-A3. Measurement procedure comparison and bias estimation using patient samples. Third Edition Wayne PA: NCCLS; 2013.
9. Horowitz GL, Altaie S, Boyd JC, Ceriotti F, Garg U, Horn P, et al. CLSI document EP28-A3. Defining, establishing, and verifying reference intervals in the clinical laboratory. Third edition Wayne PA: NCCLS; 2010.
10. McEnroe RJ, Durham AP, Goldford MD, Kondratovich MV, Lababidi S, Magari R, et al. CLSI document EP05-A3. Evaluation of precision of quantitative measurement procedure. Third edition Wayne PA: NCCLS; 2014.
11. Pierson-Perry JF, Vaks JE, Durham AP, Fischer C, Gutenbrunner C, Hiiyard D, et al. CLSI document EP17-A2. Evaluation of detection capability for clinical laboratory measurement procedures. Second edition. Wayne PA: NCCLS; 2012.
12. Tholen DW, Kroll M, Astles JR, Caffo AL, Happe TM, Krouwer J, et al. CLSI document EP06-A. Evaluation of the linearity of quantitative measurement procedures: a statistical approach. Wayne PA: NCCLS; 2003.
13. Bordeleau ME, Audemard E, Metois A, Theret L, Lisi V, Farah A, et al. Immunotherapeutic targeting of surfaceome heterogeneity in AML. Cell Rep. 2024;43(6):114260.
14. Dohner H, Wei AH, Appelbaum FR, Craddock C, DiNardo CD, Dombret H, et al. Diagnosis and management of AML in adults: 2022 recommendations from an international expert panel on behalf of the ELN. Blood. 2022;140(12):1345-77.
15. Burd EM. Validation of laboratory-developed molecular assays for infectious diseases. Clin Microbiol Rev. 2010;23(3):550-76.
16. Maiga A, Lemieux S, Pabst C, Lavallee VP, Bouvier M, Sauvageau G, et al. Transcriptome analysis of G protein-coupled receptors in distinct genetic subgroups of acute myeloid leukemia: identification of potential disease-specific targets. Blood Cancer J. 2016;6(6):e431.
